# Evaluating the efficiency loss of a double-fractured underground dam in coastal seawater intrusion mitigation under groundwater abstraction conditions

**DOI:** 10.1038/s41598-025-23771-9

**Published:** 2025-11-14

**Authors:** Asaad M. Armanuos, Martina Zeleňáková, Hany F. Abd-Elhamid, Sobhy R. Emara

**Affiliations:** 1 Irrigation and Hydraulics Engineering Department, Faculty of Engineering, Tanta University, Tanta, Egypt; 2 Institute for Sustainable and Circular Construction, Faculty of Civil Engineering, Technical University of Košice, 042 00 Košice, Slovakia; 3 Water and Water Structures Engineering Department, Faculty of Engineering, Zagazig University, Zagazig, 44519 Egypt; 4 Department of Environmental Engineering, Faculty of Civil Engineering, Technical University of Košice, 042 00 Košice, Slovakia

**Keywords:** Seawater intrusion, Coastal aquifer, Sustainability, Groundwater resources, Environmental sciences, Hydrology

## Abstract

Seawater intrusion (SWI) poses a continuing threat to the sustainability of groundwater resources in coastal aquifers, especially in areas where demand for freshwater is high. Among the various engineering approaches, underground dams as physical barriers are commonly used to restrict the inland migration of seawater into coastal aquifers. However, their effectiveness can be significantly affected by structural design and groundwater extraction practices. This research implements the SEAWAT numerical code to investigate the performance of a double-fractured underground dam across changing hydrological conditions. The analysis emphasises the influence of fracture aperture and height, underground dam depth and location and the abstraction well depth, location and abstraction rate. Two representative case studies were analysed: the Henry problem, serving as a benchmark, and the Akrotiri coastal aquifer in Cyprus, demonstrating a real-world case study. The outcomes show that dam efficiency decreases significantly when underground dams are located closer to the seawater boundary, or when dam fractures are positioned close to the base of the aquifer. High pumping rates and a location of the well near the seawater-freshwater interface increase the loss of efficiency, while high saltwater density exacerbates these impacts. The study also demonstrates that dam location has a greater effect on efficiency than its depth. Overall, the outcomes highlight the lack of specific design criteria and strategies for carefully considering groundwater abstraction and the long-term importance of underground dams for coastal groundwater management. The findings provide practical insights for coastal aquifer management, offering guidance for more sustainable utilisation of groundwater resources in vulnerable coastal regions.

## Introduction

Arid and semi-arid regions are expected to be the dominant land use on the Earth in the future, with diminishing surface water supplies due to pollution, increasing agricultural requirements and increased economic and demographic growth. Thus, groundwater will be put under greater quantitative and qualitative stress, leading to its faster depletion. Coastal regions already exposed to tremendous socioeconomic and environmental stress will suffer the most, with climate change contributing most notably to the issues^1–5^. This will have a very important impact in semi-arid and deltaic areas, where human populations are high and sustainable water management is necessary in order to balance social, economic and environmental needs^6^. One of the most serious issues in coastal areas is the salinisation of water supplies caused by both human and natural factors. The growing demand for freshwater, already a problem and likely to be compounded by climate change and land use changes, has generated interest in groundwater salinisation^7^. Groundwater is increasingly seen as a significant water source because it is less prone to contamination in comparison with surface water and because it has a vast capacity for storage^8,9^.

Seawater intrusion occurs when saltwater seeps into coastal aquifers and contaminates freshwater supplies. Such aquifers are located in a complex environment shaped by fluctuating water volumes, various salinity levels and inhomogeneous hydraulic properties. The interaction among water density, salinity, climatic fluctuations, groundwater extraction and sea level rise creates complex hydrologic conditions and impacts the distribution of dissolved salts. Small-scale phenomena exert strong impacts on coastal water systems; hence, seawater intrusion is a cause for concern^10,11^. Groundwater depletion, or excessive use, may be viewed in two ways. The first is merely a reduction in the overall water level in the zone of saturation with no consideration for water quality. The second reduction in the freshwater available in an aquifer is worse. Seawater intrusion may severely affect water quality but not necessarily severely reduce the overall amount of groundwater. It is difficult to measure and track depletion due to the lack of geologic data and the time needed to make the estimates. Having multiple causes and widespread effects, groundwater depletion is difficult to fully evaluate^12^.

Everywhere in the world, coastal aquifers are stressed with the depletion of groundwater resources brought about by expanding populations and developing industries. With about 40% of the world’s population living in coastal zones^13^, seawater intrusion is now a problem with increasing urgency. Its effects not only concern water resources but extend to the health of the population, the economy and even the culture of the places. Because of the problem’s severity, it has spawned a body of research on the need for sustainable water solutions. The study of saltwater intrusion dates back to Henry^14^, who developed a steady-state quasi-analytical model for a hypothetical unconfined coastal aquifer. His model, which described freshwater flowing from an inland boundary toward the sea while seawater moved inland, became a cornerstone for future research. The aquifer in his study measured just 2 m in length, depth and width, but despite its simplicity, it provided valuable insights into the movement of saltwater in coastal groundwater systems. Since then, many researchers have built upon Henry’s work, either modifying his original model or using it as a benchmark to test their numerical simulations^15–20^.

Physical barriers in the form of concrete walls, grouting injections, bentonite layers, slurry walls and sheet piles are commonly utilised along shores to prevent seawater intrusion. Their effectiveness in slowing and controlling saltwater seepage into freshwater aquifers has been shown through studies^21^. For example, Galeati et al. ^22^ used a 2D simulation to demonstrate the positive impact of such barriers in southern Italy. Similarly, Sugio et al. ^23^ simulated cement grouting in the Okinawa-Jima Island of Japan using experimental sand-box testing and numerical simulations, like finite difference modelling. Nishikawa et al.’s ^24^ model of the Dominguez Gap coastal area of Los Angeles using the USGS-developed SUTRA code has corroborated such results.

An important practical application is the Komesu underground concrete dam in Japan. The huge cutoff wall measuring 2,320 m long, 0.54 m wide and 70 m below the mean sea level in depth successfully separated an aquifer from saltwater intrusion^25^. Apart from this, research carried out by Abdoulhalik and Ahmed^26^ has demonstrated that the most efficient deep physical barriers in stopping the intrusion of saltwater into freshwater reservoirs are those placed nearer the coastline and ahead of the toe of the seawater intrusion. Onder and Yilmaz^27^ discussed various types of underground dams and the methods and design for constructing and creating such dams, as well as the importance they play in sustainable water management. The efficiency of such dams in preventing SWI and controlling groundwater flow was analysed using numerical simulations with the use of the MODFLOW model. The findings indicate that not only do underground dams prevent saltwater intrusion; they also increase the capacity of an aquifer to store water, promoting long-term water resource sustainability.

Tsanis and Song^28^ simulated seawater intrusion in the Upper Florida Aquifer in South Carolina through utilisation of the SUTRA model. The study found that injection wells would be effective in slowing intrusion but were restricted to confined aquifers. Ebeling et al. ^29^ developed a 2D SEAWAT and FloPy model of seawater intrusion to compare a single pumping barrier and a mixed hydraulic barrier. Their research helped determine the most effective management scheme in mitigating saltwater intrusion. Abdoulhalik et al. ^30^ also introduced a hybrid physical barrier system that includes a semi-permeable underground dam and an impermeable cutoff wall. The new method uses freshwater to push saltwater back to the sea, minimising the intrusion zone in coastal aquifers. Their research also addressed the function of anti-seepage walls in blocking saltwater intrusion in multiphase aquifers.

Kaleris and Ziogas^31^ used numerical simulations to analyse the impact of cutoff walls on seawater intrusion and the degree to which they secure groundwater extraction along a shoreline. Their analysis found the efficiency of the walls to be a function of factors such as the depth of the wall, distance from the shore, velocity ratios, mixing intensity, conductivity variations and relative permeability. It also found that cutoff walls offered better protection for individual wells compared to drainage systems. The walls are most effective where the extraction of groundwater is done near the shoreline, in higher depths and in aquifers with low velocity ratios, low mixing intensity and high heterogeneity.

Chang et al. ^32^ studied the use of underground dams with the minimum effective height required to prevent seawater intrusion. Using experimental tests and simulations, they concluded that the maximum fresh groundwater outflow occurs with the minimum dam height. Their analysis also revealed that the minimum effective dam height is lower than the height of seawater intrusion in the absence of a dam. The minimum dam height required and the maximum fresh groundwater outflow both increased with an increase in the distance of the dam from the coastline. Al-Taliby and Dekhn^33^ conducted a study using a three-dimensional SEAWAT model to explore how groundwater pumping from different wells affects saltwater intrusion and submarine groundwater discharge in coastal aquifers. Their findings showed that both well placement and extraction rates influence how far saltwater moves inland. When pumping rates increased from 0.165 m³/s to 0.53166 m³/s, the saltwater wedge advanced significantly, extending from 589 m to 1319 m. However, a well located closer to the coastline altered the flow dynamics, sometimes forcing the saltwater wedge to retreat back toward the sea, effectively acting as a barrier to further intrusion.

Ozaki et al. ^34^ carried out a lab-scale experiment to examine how a barrier well affects freshwater flow from a production well. Alongside their physical tests, they created a 2D numerical model that mimicked the same conditions used in the experiments. Their findings showed that when the abstraction ratio dropped below a certain threshold, saltwater began creeping toward the barrier well, increasing salinity levels in the system.

Wu et al. ^35^ used 3D numerical simulations to compare subsurface dams, cutoffs and fully penetrating barriers to decrease seawater intrusion in barrier-only systems, finding that cutoffs tended to outperform subsurface dams in this application while still allowing for elevated safe pumping rates when optimally designed. Fully penetrating barriers are always optimally performed in all cases. Extending this work, Wu et al. ^36^ developed an analytical method, calibrated to numerical simulations, to predict effectively the width of the seawater wedge as well as the maximum safe extraction rates in aquifers with fully penetrating, finite-length barriers. Analyses by these researchers demonstrated that aquifer conditions strongly affect the optimal placement location when minimising seawater intrusion, while barrier length as well as distance from wells dominate when maximising extraction. Supplementing these analyses, Wu and Lu ^37^ investigated residual saltwater persistence upstream from subsurface dams under diverse conditions, demonstrating that groundwater level fluctuations between seasons increase dilution as well as quicken removal when compared to steady-head scenarios while still taking decades to complete desalinisation.

Laabidi et al. ^38^ recently used FEFLOW to examine how fractures in concrete cutoff walls impact their ability to stop SWI in coastal aquifers. To assess the performance of fractured barriers, they compared the lengths of the SWI wedge after fracturing with the base Henry case. Their findings revealed that the efficacy of a cutoff wall is highly sensitive to factors such as fracture aperture, aperture height and saltwater density. Armanuos et al. ^16^ conducted a study using the SEAWAT code to explore how groundwater abstraction influences the efficiency of damaged underground dams with a single fracture in limiting seawater intrusion in coastal aquifers. The results showed that factors such as well positioning, pumping rates and fracture properties significantly affect how well a single fractured underground dam can control saltwater intrusion.

This study investigates the influence of structural fractures in underground dams on their effectiveness in mitigating saltwater intrusion into coastal aquifers. Numerical simulations were conducted using the SEAWAT code to compare saltwater movement in scenarios with both intact and double-fractured dams, highlighting the impact of structural integrity on dam performance. Model validation was achieved through benchmark testing using the Henry problem^14^ and a real world case study of the Akrotiri coastal aquifer in Cyprus^39^. The analysis further explores the effects of varying groundwater abstraction rates, comparing them to a baseline scenario to assess their influence on dam efficiency. Key parameters, such as dam height, position, fracture size and location, were systematically examined to evaluate their roles in controlling saltwater intrusion. A sensitivity analysis was also performed to assess the resilience of fractured dams under diverse hydrogeological conditions. The real-world impact of fractures on underground dams under actual field conditions is investigated for the first time ever, emphasising the need for a reliable physical barrier construction strategy. By examining the interplay between structural damage and groundwater extraction, the study offers critical insights for optimising the design and management of underground dams in coastal aquifer systems.

## Materials and methods

### Numerical modelling

The effect of groundwater abstraction on a double-fractured concrete underground dam’s ability to mitigate SWI in unconfined coastal aquifers was investigated using SEAWAT^40^. In order to provide important general conclusions, the influence of a double-fractured concrete underground dam was also simulated using Henry’s issue configurations with the same variables, fresh groundwater and seawater border conditions^14^. The SEAWAT code’s input parameter settings for the Henry saltwater problem utilised in numerical simulations are tabulated and presented in Table 1. The SEAWAT code has become commonly used for numerical simulation of saltwater intrusion. The code that connects MODFLOW with MT3DMS is called SEAWAT. The code is used to solve solute transport equations as well as the associated groundwater flow equations. The SEAWAT code uses variable density groundwater flow calculations. The influence of freshwater abstraction on the efficiency of a double-fractured underground dam was examined in this study using the dimensions of the Henry saltwater problem.

### Examined cases

The developed SEAWAT model was employed to simulate the application of a double-fractured concrete underground dam in coastal aquifers. To ensure a consistent evaluation, two scenarios were analysed: a benchmark case using the Henry problem and a real-world case involving a double-fractured concrete underground dam installed in a field-scale cross section of the Akrotiri coastal aquifer in Cyprus.

#### A hypothetical case study (Henry problem)

**Fig. 1:**
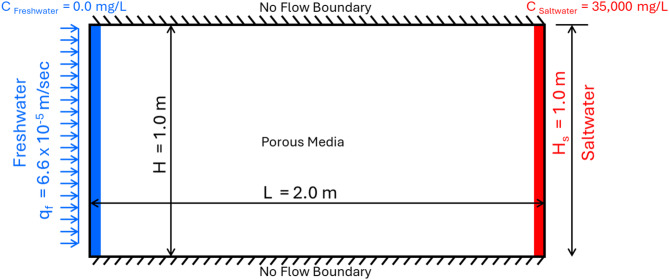
Henry’s problem domain and boundary conditions.

The sizes of the generated model domain are 100 cm in vertical coordinates and 200 cm in horizontal coordinates. The cell sizes were set to 2.0 cm (∆x = ∆y). For every simulated case, the longitudinal dispersivity and the transversal dispersivity values were set to 1 and 0.1 mm, correspondingly. The seawater head (h_s_) on the seaside was set at 100 cm, while the flux of the freshwater on the freshwater boundary was fixed at 6.6 × 10^− 5^ m/s. Considering a TDS concentration (salt concentration) of 35,000 mg/L, the density of the seawater on the seawater boundary side was determined to be 1025 kg/m^3^ (Fig. 1). The freshwater within the freshwater boundary had a TDS concentration (salt concentration) of 0.0 mg/L and a density of 1000 kg/m^3^. The porous aquifer media’s initial salinity content was fixed at 0.0 mg/L. The aquifer hydraulic conductivity was 0.01 m/s in all three directions, as it was presumed that the aquifer had been homogeneous and isotropic with porosity of 0.35. The definitions of the parameters of the solved problem and numerical simulations are displayed in Table 1. The upper and lower fracture apertures were adjusted in this study to range from 0.005 to 0.0125 m; these values are compatible with previous studies for fractures in concrete cutoff and underground dams: Cermak et al. ^41^ (from 0.0005 to 0.0015 m), Sato et al. ^42^ (from 0.01 to 0.053 m), Laabidi et al. ^38^ (from 0.001 to 0.006 m) and Armanuos et al. ^16^ (from 0.005 to 0.01 m). The height of the upper and lower fractures was adjusted to range from 0.3 to 0.7 and is compatible with previous studies: Laabidi et al. ^38^ (from 0.16 to 0.67) and Armanuos et al. ^16^ (from 0.1 to 0.2).

**Table 1:** Parameters of the numerical simulation.

Symbol	Definition	Value	Unit
L	Aquifer length	2.0	m
d	Aquifer depth	1.0	m
n	Aquifer porosity	0.35	-
k	Aquifer hydraulic conductivity	0.01	m/sec
d_o_	Value of the coefficient of molecular diffusion	6.6 × 10^-6^	m^2^/sec
q_b_	Flux of the freshwater boundary	6.6 × 10^-5^	m/sec
$\:{{\uprho\:}}_{\text{f}}$	Freshwater boundary density	1000	Kg/m^3^
$\:{{\uprho\:}}_{\text{s}}$	Seawater boundary density	1025	Kg/m^3^
$\:{\text{C}}_{\text{f}}$	Concentration of the freshwater	0.0	mg/l
$\:{\text{C}}_{\text{s}}$	Concentration of the seawater	35,000	mg/l
$\:{{\upalpha\:}}_{\text{l}}$	Dispersivity coefficient in the longitudinal direction	1.0	mm
$\:{{\upalpha\:}}_{\text{t}}$	Dispersivity coefficient in the transversal direction	0.1	mm
g	Gravitational acceleration	9.81	m/s^2^
$\:{\upmu\:}$	Viscosity of the fluid	0.001	Kg/m.s
$\:\varDelta\:\text{z}$	Dimension of the cell in the vertical direction	0.02	m
$\:\varDelta\:\text{x}$	Dimension of the cell in the horizontal direction	0.02	m
$\:{L}_{d}$	Dam location measured from the saltwater boundary	12.8, 25.6, 38.4 and 51.2	cm
$\:{X}_{d}/{L}_{o}$	Ratio of the dam location	0.2, 0.4, 0.6 and 0.8	-
H_fu_	Height of the upper fracture	0.25, 0.30, 0.50 and 0.60	m
H_fl._	Height of the lower fracture	0.25, 0.30, 0.40 and 0.50	m
D_fu_	Diameter of the upper fracture	0.50, 0.75. 1.0 and 1.25	cm
D_fl._	Diameter of the lower fracture	0.50, 0.75. 1.0 and 1.25	cm
H_d_	Height of the dam	0.50, 0.60, 0.70 and 0.80	m
L_w_	Location of the abstraction well	51.2, 64, 76.8 and 89.6	m
H_w_	Height of the abstraction well	12, 24, 36 and 48	m
X_w_/X_d_	Ratio of well location	0.80, 1.0, 1.20 and 1.40	-
H_w_/H_d_	Ratio of well depth	0.2, 0.4, 0.6 and 0.8	-

#### Real case study (Akrotiri coastal aquifer in Cyprus)

The SEAWAT code was used to simulate saltwater intrusion within the Akrotiri coastal aquifer in Cyprus. The model was used to investigate both the overall response of SWI and the performance of an underground dam, specifically in cases where double fractures would impact its operation. The aquifer occurs at Cyprus’s southernmost peninsula, west of Limassol.

**Fig. 2:**
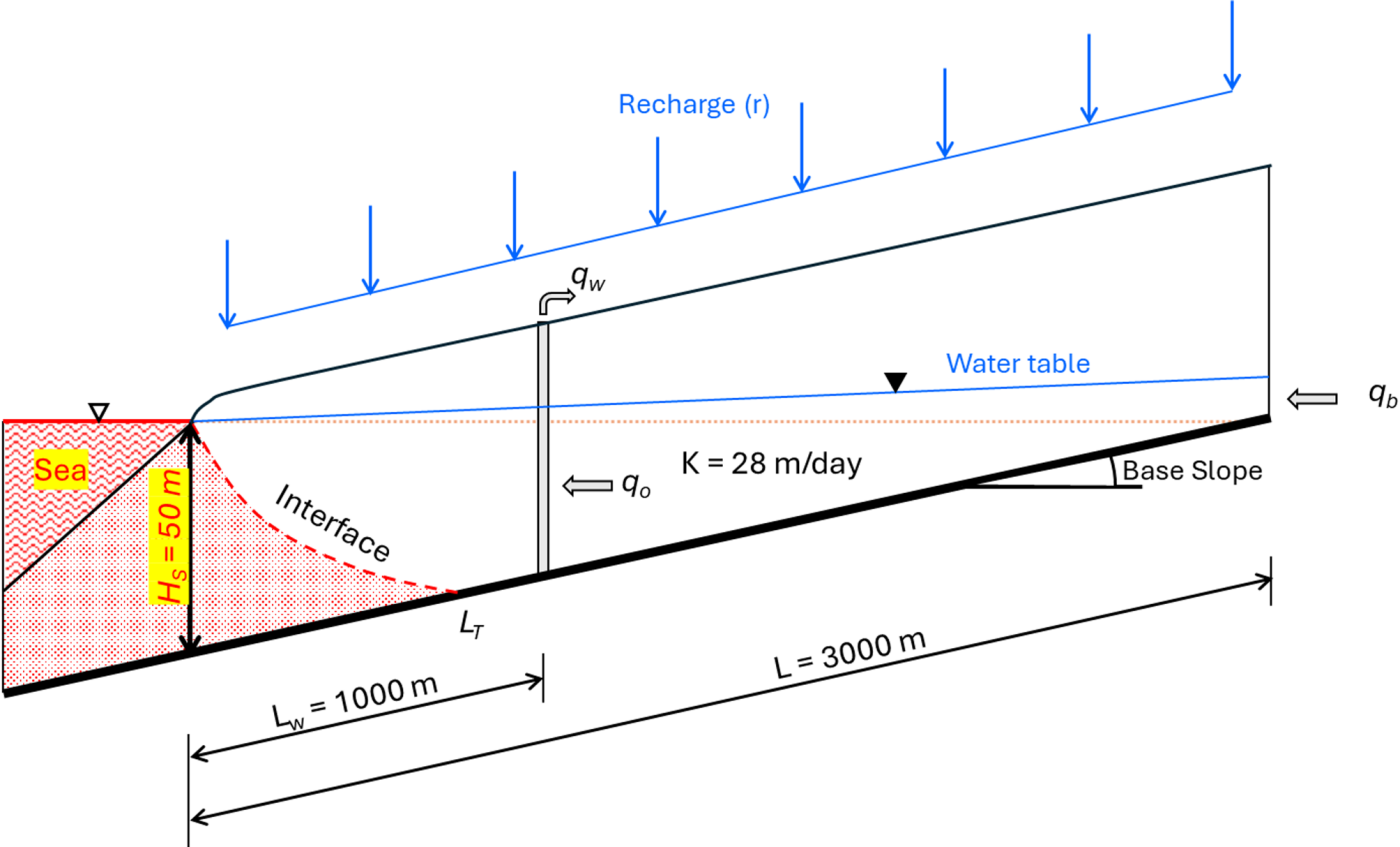
Two-dimensional concept of the Akrotiri aquifer.

Figure 2 shows the conceptual hydrogeologic profile of the aquifer system. An explicit three-dimensional representation of the Akrotiri aquifer was built using SEAWAT. The model domain was 3,000 m long and had a depth of 100 m. Each model cell was dimensioned at 6 × 6 m both in the horizontal (X) and vertical (Z) axes. Key physical parameters were saltwater height at 50 m and bed slope at 1.7%. The aquifer receives an annual recharge of around 83 mm. Hydraulic properties and SEAWAT input parameters, tabulated in Table 2, where the hydraulic conductivity of 28 m/day and the specific yield at 0.2 were used. Freshwater and saltwater densities were fixed at 988 kg/m³ and 1,024 kg/m³, correspondingly. The model was executed at steady state using a recharge of 83 mm/year. The inland boundary flow was set at 314 m³/year/m of aquifer width^39^.

**Table 2:** Numerical model input for the Akrotiri coastal aquifer, Cyprus.

Parameter	Value	Unit
Inflow from the inland boundary	314	m³/year/m
Hydraulic conductivity	28	m/day
Mean aquifer yield	0.2	-
Length of the aquifer	3000	km
Depth of the aquifer at the coastline	50	m
Pumping location (Lw)	1000	m
Pumping rate	235	m³/year/m
Natural recharge	83	mm/year
Density of the freshwater	988	kg/m³
Density of the saltwater	1024	kg/m³
Freshwater concentration	0	mg/L
Saltwater concentration	35,000	mg/L
Dispersivity in the longitudinal direction	2	m
Dispersivity in the transverse direction	2	m
Dimension of the cell	6 × 6	m
Aquifer width	1	m
Toe location	979.2	m
Dam location measured from the saltwater boundary	200, 300, 400, 500	m
The ratio of the dam location	0.2, 0.3, 0.4, 0.5	-
Height of the dam	25, 28, 31, 34	m
Height of the upper fracture	20	m
Height of the lower fracture	6	m
Diameter of the upper fracture	0.1	m
Diameter of the lower fracture	0.1	m

### Dimensional analysis

The dimensional analysis of the present research can be stated as follows:

1 $$\:\varnothing\:\left(R,K,\:H,\:{H}_{d},\:{H}_{uf},\:{H}_{lf},\:{D}_{uf},\:{D}_{lf},{{H}_{w},{L}_{o},\:X}_{d}{,X}_{w},\:{Q}_{w},\:{Q}_{f},\:{\rho\:}_{f},\:{\rho\:}_{s},\:g,\:n,{v}_{s}{,\:{v}_{f},\:C}_{s},\:{C}_{f},\:{\alpha\:}_{l},\:{\alpha\:}_{T}\right)=0.0$$

The total number of variables = 24, the total number of repeated dimensions = 3, and the number of π = 21.

Select the following parameters H, K and $\:{\rho\:}_{s}$:

2 $$\:\varnothing\:\left({\pi\:}_{1},{\pi\:}_{2},{\pi\:}_{3},{\pi\:}_{4},{\pi\:}_{5},{\pi\:}_{6},{\pi\:}_{7},{\pi\:}_{8},{\pi\:}_{9},{\pi\:}_{10},{\pi\:}_{11},{\pi\:}_{12},{\pi\:}_{13},{\pi\:}_{14},{\pi\:}_{15},{\pi\:}_{16},{\pi\:}_{17},{\pi\:}_{18},{\pi\:}_{19},{\pi\:}_{20},{\pi\:}_{21}\right)=0.0$$

3 $$\:\varnothing\:\left(R,\frac{{H}_{d}}{H},\:\frac{{H}_{uf}}{H},\:\frac{{H}_{lf}}{H},\:\frac{{D}_{uf}}{H},\:\frac{{D}_{lf}}{H},\frac{{H}_{w}}{H},\frac{{L}_{o}}{H},\frac{{X}_{d}}{H},\:\frac{{X}_{w}}{H},\:\frac{{Q}_{f}}{{H}^{2}K},\frac{{Q}_{w}}{{H}^{2}K},\:\frac{{\rho\:}_{f}}{{\rho\:}_{s}},\:g,\:n,\frac{{v}_{s}}{HK},\frac{{v}_{f}}{HK},\:\frac{{C}_{f}}{{\rho\:}_{s}},\:\frac{{C}_{S}}{{\rho\:}_{s}},\:{\alpha\:}_{l},\:{\alpha\:}_{T}\right)=0.0$$

In the present research, the following parameters are considered to be constant: k, H, L_o_, g, n,α_L_, α_T_, v_s_, v_f_, C_s_, and C_f_ .

The combined impact of the upper fracture height H_uf_ and the underground dam height H_d_ can be studied when π_3_ is divided by π_2_.

The combined impact of the lower fracture height H_lf_ and the underground dam height H_d_ can be studied when π_4_ is divided by π_2_.

The combined impact of the upper fracture aperture D_uf_ and the underground dam height H_d_ can be studied when π_5_ is divided by π_2_.

The combined impact of the lower fracture aperture D_lf_ and the underground dam height H_d_ can be studied when π_6_ is divided by π_2_.

The combined impact of the abstraction well height H_w_ and the underground dam height H_d_ can be studied when π_7_ is divided by π_2_.

To study the effect of underground dam distance X_d_ and initial length of seawater intrusion wedge L_o_, π_9_ is divided by π_8_.

The combined impact of the well distance X_w_ and the underground dam distance X_d_ can be studied when π_10_ is divided by π_9_.

The combined impact of the abstraction well rate Q_w_ and the freshwater flux Q_f_ can be studied when π_12_ is divided by π_11_.

Consequently, we can gain the subsequent function for the saltwater intrusion wedge length repulsion ratio:

4 $$\:R=f\left(\frac{{H}_{d}}{H},\:\frac{{H}_{uf}}{{H}_{d}},\:\frac{{H}_{lf}}{{H}_{d}},\:\frac{{D}_{uf}}{{H}_{d}},\:\frac{{D}_{lf}}{{H}_{d}},\frac{{H}_{w}}{{H}_{d}},\frac{{X}_{d}}{{L}_{o}},\:\frac{{X}_{w}}{{X}_{d}},\:\frac{{Q}_{w}}{{Q}_{f}}\right)$$

### Investigated configurations

**Fig. 3:**
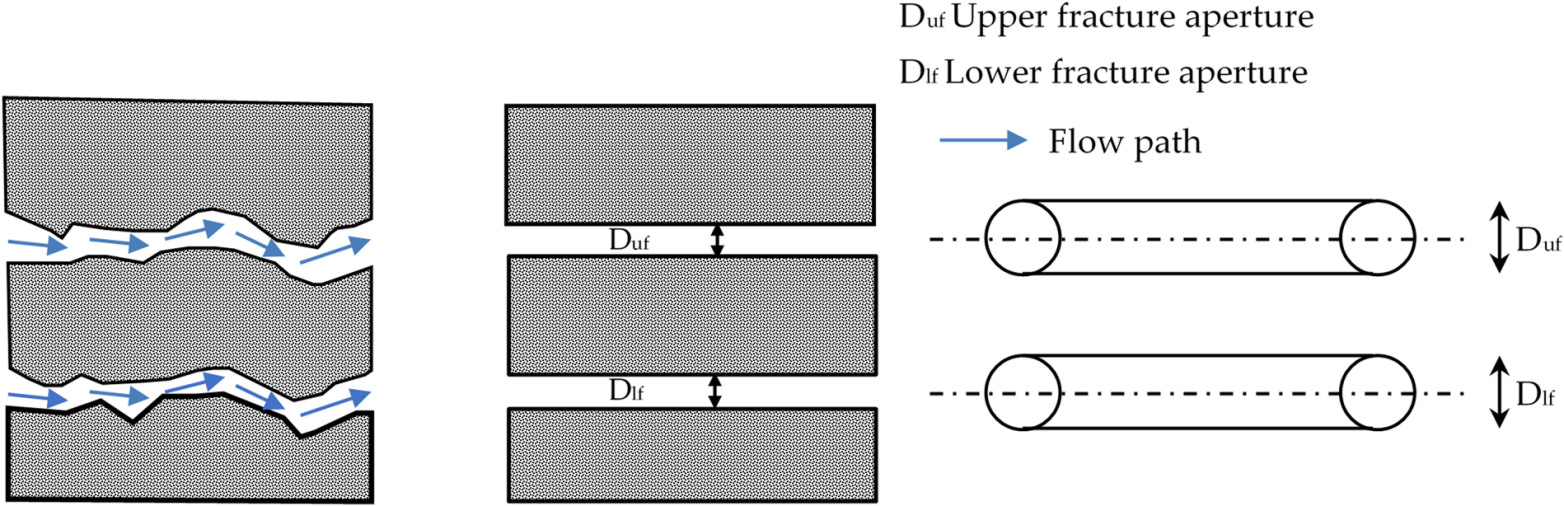
Actual, simulated and idealised fracture forms in an underground dam.

A concrete structure should have both structural and resistive purposes. In this situation, the stability, lifespan and waterproofing of the structure depend heavily on the cracking of the reinforced concrete. Concrete structural degradation frequently manifests as fractures, cracks, scaling, weathering and spalling (Fig. 3). Rombach and Faron^43^ stated that the cracks and fractures may be oblique or horizontal in orientation. Many things can lead to this damage; however, the following are the most frequent ones:

Concrete can crack due to several mechanisms. Plastic shrinkage occurs in new, unset concrete during evaporation of excess water and leaves voids. Freezing and thawing in cold climates expands pore water, creating pressure, and cracking. Carbonation decreases the pH of pore solution, leading to steel corrosion, loss of strength in hydrate products, and fissuring. Settlement cracking, expanding over time, can be due to inadequate support in soil, and reinforcement corrosion produces volumetrically expanding rust, spalling, and cover cracking. Chemical attack can alter volume, diminish material strength, and, in severe instances, cause failure. Overloading or cyclical load can put in excess of design capacity and cause crack or collapse. Lastly, fire damage produces thermal gradients, leading to surface and microcracking, loss of strength, and an increase in susceptibility to subsequent deterioration.

### Sensitivity analysis

**Fig. 4:**
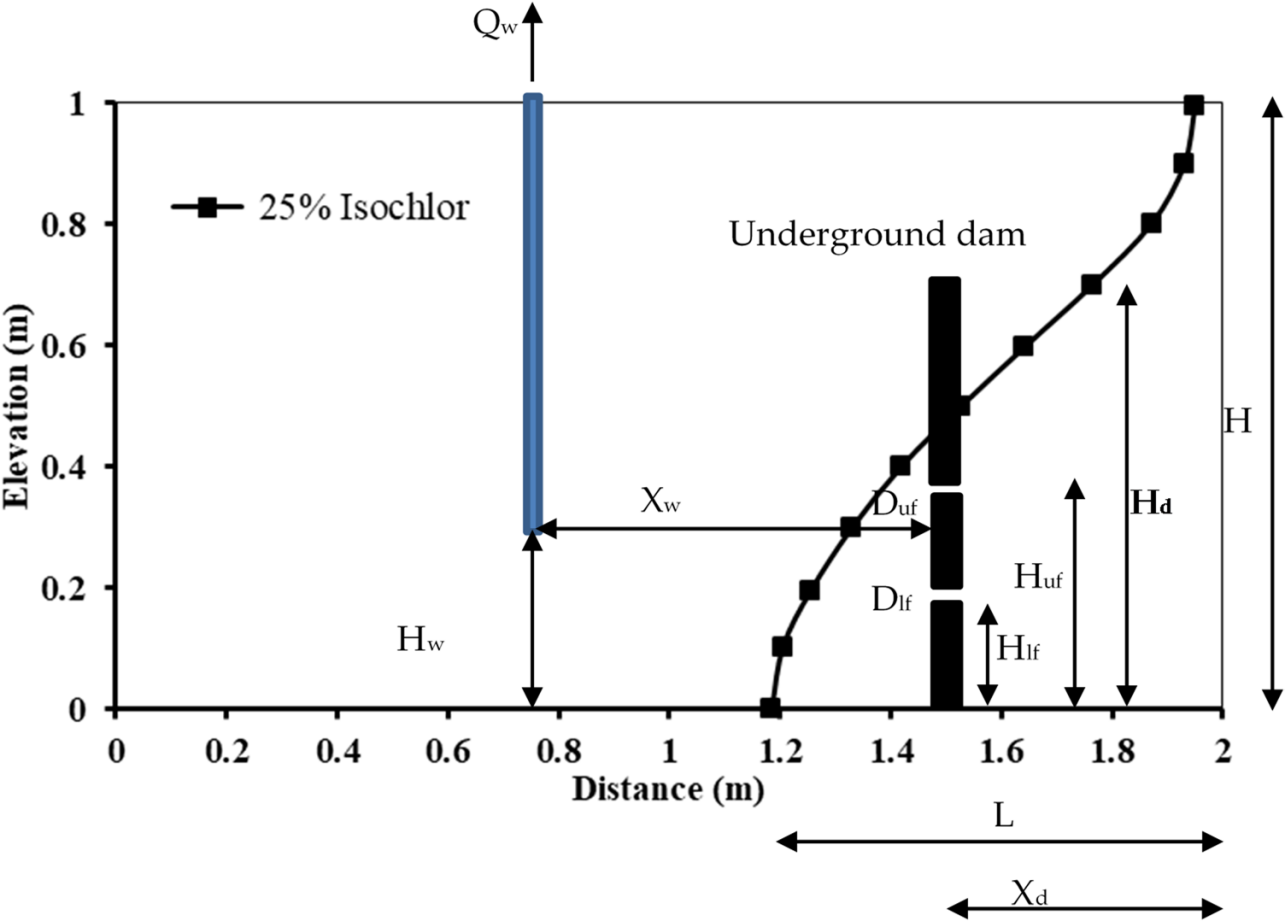
Schematic illustration of the primary problem variables and the simulated numerical setups.

Using the precise geometric properties of the Henry problem, a sensitivity analysis was carried out to assess the effect of groundwater extraction on the length of seawater intrusion penetration and the effectiveness of the double-fractured concrete underground dam in preventing seawater intrusion in coastal aquifers (Fig. 4). The following factors are being tested: the rate at which freshwater is extracted from the groundwater aquifer, the height of the well, the ratio of well location, the underground dam location, the underground dam height, the diameter of the upper fracture, the diameter of the lower fracture, the upper fracture height, the lower fracture height and the seawater density.

The key parameters utilised in the sensitivity study simulations are shown in Table 3 and in Fig. 4. The tested numerical simulations presented use the intruded length of the seawater intrusion of the case reference scenario for the Henry saltwater problem.

**Table 3:** Definitions of parameters utilised for conducting the numerical simulation.

Parameter	Definition
H_d_	The underground dam height measured from the bottom of the aquifer
L_d_	The distance between the underground dam location and the saltwater boundary
H_w_	The well screen height calculated from the bottom of the aquifer
L_w_	The horizontal distance between the underground dam and the well location
D_fu_	The diameter of the upper fracture
D_fl._	The diameter of the lower fracture
H_fu_	The upper fracture height measured from the aquifer base
H_fl._	The lower fracture height measured from the aquifer base
Q_w_	The groundwater well rate of abstraction
L_toe0_	The saltwater intrusion wedge length for the base case
L_toew_	The saltwater intrusion length of the seawater after implementing the underground dam
L_toedfw_	The seawater intrusion wedge length with respect to the double fracture in the underground dam and the fresh groundwater extraction
R_Ew/0_	Seawater repulsion percentage with respect to the underground dam construction comparing with the base case scenario of the Henry seawater intrusion problem: (L_toe0_ − L_toew_)/L_toe0_
R_Eadfw/0_	Saltwater repulsion percentage caused by the extraction of fresh groundwater near the double-fractured underground dam comparing with the base case: (L_toe0_ − L_toefw_)/L_toe0_.
R	The saltwater intrusion wedge length repulsion ratio
K	The hydraulic conductivity value of the aquifer
H	The depth of the aquifer
ρ_s_	The seawater density
ρ_f_	The freshwater density
g	The value of gravity acceleration
n	The aquifer’s medium porosity
C_s_	The concentration of saline water
C_f_	The concentration of the freshwater
v_s_	The seawater viscosity
v_f_	The freshwater viscosity
α_L_	The dispersivity value in the longitudinal direction
α_T_	The dispersivity value in the transversal direction

### Governing equations

The variable-density flow in terms of freshwater heads and the solute movement in groundwater, as used in SEAWAT, can be obtained from the research conducted by Guo and Langevin^40^. Binet et al. ^44^ investigated the exchanges among a conduit network and a solid matrix using a 2D comparable porous medium. The conduit was modelled via a discrete element, and flows were calculated using Manning–Strickler’s law. It was demonstrated that flows in karstic and fractured conduits could be adequately described by Manning–Strickler’s law.

A simple Darcy’s law, the Hagen–Poiseuille law or Manning–Strickler’s law might be used to explain the conductivity tensor K_ij_ of the Darcian velocity equation^45^.

5 $$\:{Q}_{c}=-{A}_{c}.f.{r}^{\frac{2}{3}}.\sqrt{\frac{dh}{dx}}\:\:$$

dh/dx is the fracture’s head loss at the x-coordinate [dimensionless]; r is the hydraulic radius of the fracture [L]; f is the friction coefficient [L^1/3^T^−1^]; and A_c_ is the area of the fracture [L^2^].

## Results and discussion

### A hypothetical case study (Henry problem)

#### Model calibration

**Fig. 5:**
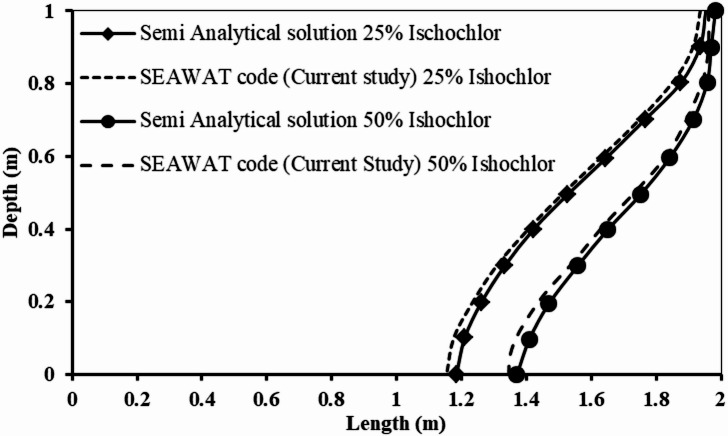
A comparison of the seawater intrusion wedge as modelled by the SEAWAT code with the semi-analytical solution of the Henry seawater intrusion problem.

Utilising a semi-analytical solution and the SEAWAT code, Fig. 5 illustrates the seawater intrusion wedge for the Henry problem. The salt concentration contour line of 7850 mg/L (25% Isochlor) and the salt concentration contour line of 17,500 mg/L (50% Isochlor) showed excellent agreement, according to the analysis comparing the two values. For a salt content of 17,500 mg/L, the saltwater intrusion penetrating is 63 cm for the semi-analytical solution and 65.7 cm for the solution of the SEAWAT code. Furthermore, at a salinity content of 7850 mg/L, the seawater intrusion penetrates 84.7 cm for the semi-analytical solution and 81.5 cm for the SEAWAT code solution. For salt concentrations of 17,500 and 7850 mg/L, accordingly, the absolute error of the saltwater intrusion wedge penetration length among the semi-analytical solutions along with the SEAWAT code is 2.7 cm and 3.2 cm, respectively.

**Fig. 6:**
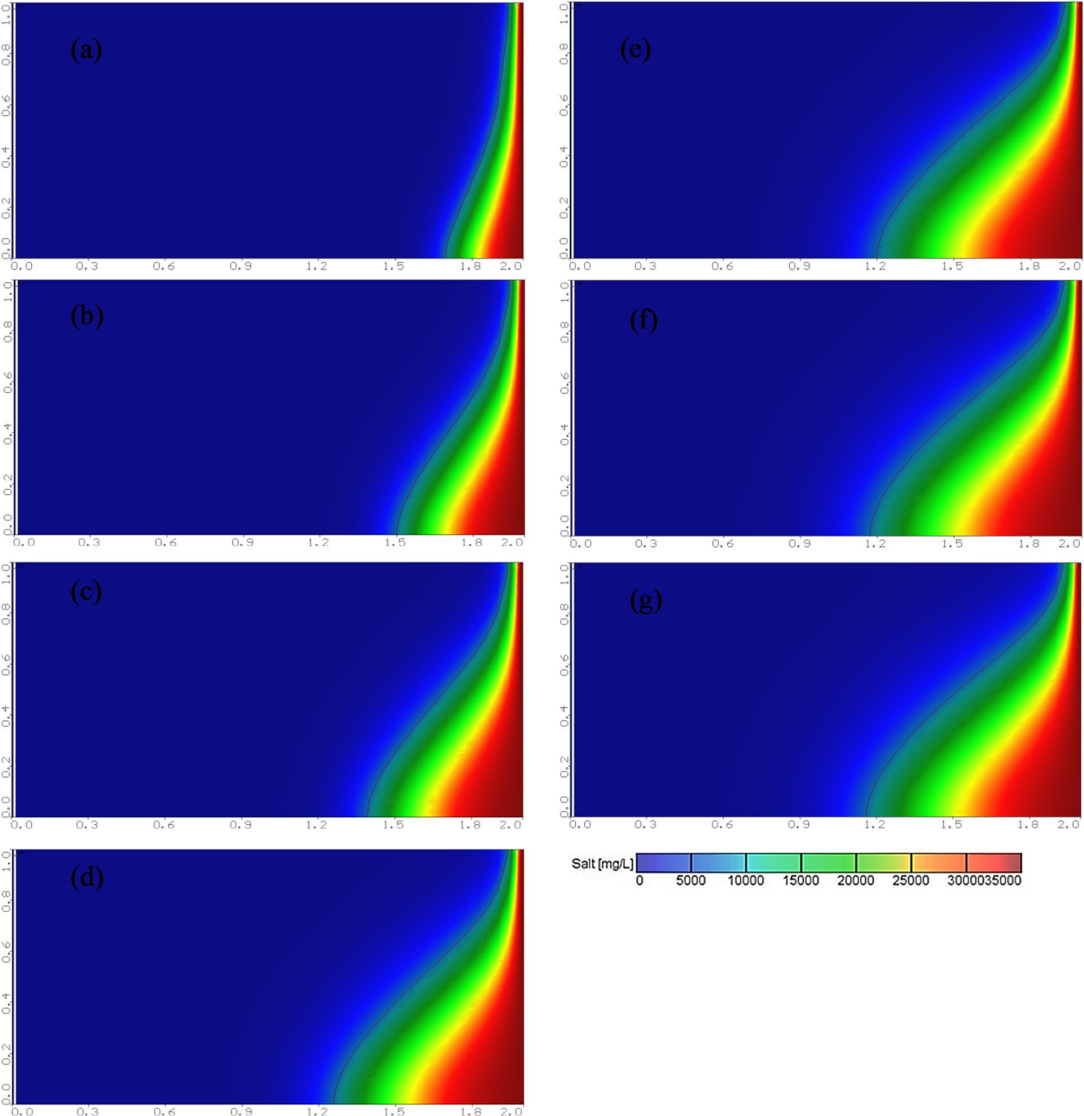
Evolution of the interface between salt and fresh water over time at: (**a**) 5, (**b**) 15, (**c**) 25, (**d**) 50, (**e**) 75, (**f**) 100 and (**g**) 125 min, dimension in meters, black line indicates 25% isohaline concentration.

Figure 6 displays a set of diagrams showing increased saltwater intrusion interference with time. The seawater interference wedge appears in Henry’s problem at different time intervals: 5, 15, 25, 50, 75, 100, and 125 min, until the interference reached a distance of 65.7 cm inland representing the steady state.

**Fig. 7:**
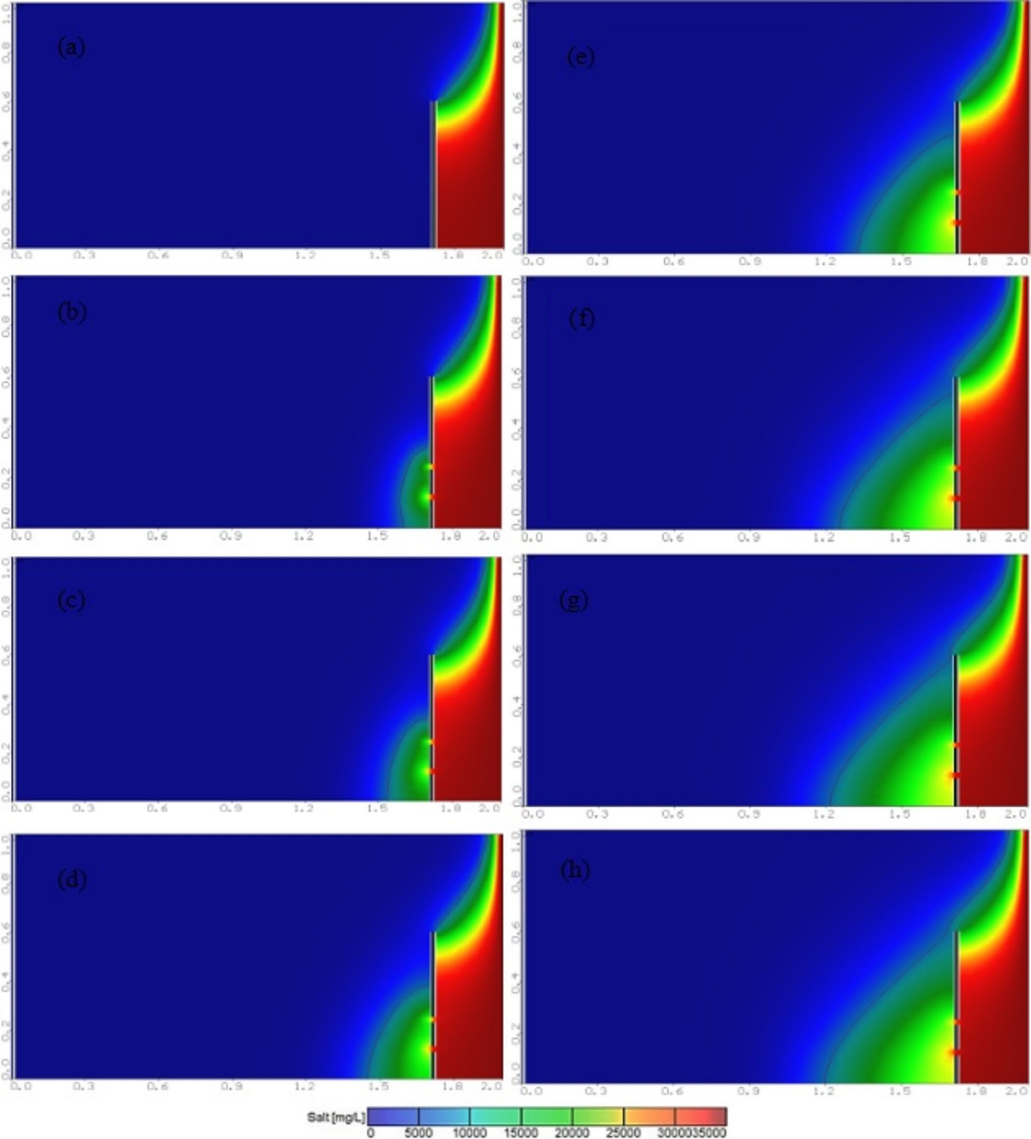
Evolution of the interface between salt and fresh water over time at: (a) 5, (b) 15, (c) 25, (d) 50, (e) 75, (f) 100 and (g) 125 min for the case (X_d_/L_o_=0.4, H_d_=50 cm & X_w_/X_d_=1.0 & Q_w_=10%Q_f_), dimension in meters, black line indicates 25% isohaline concentration.

**Fig. 8:**
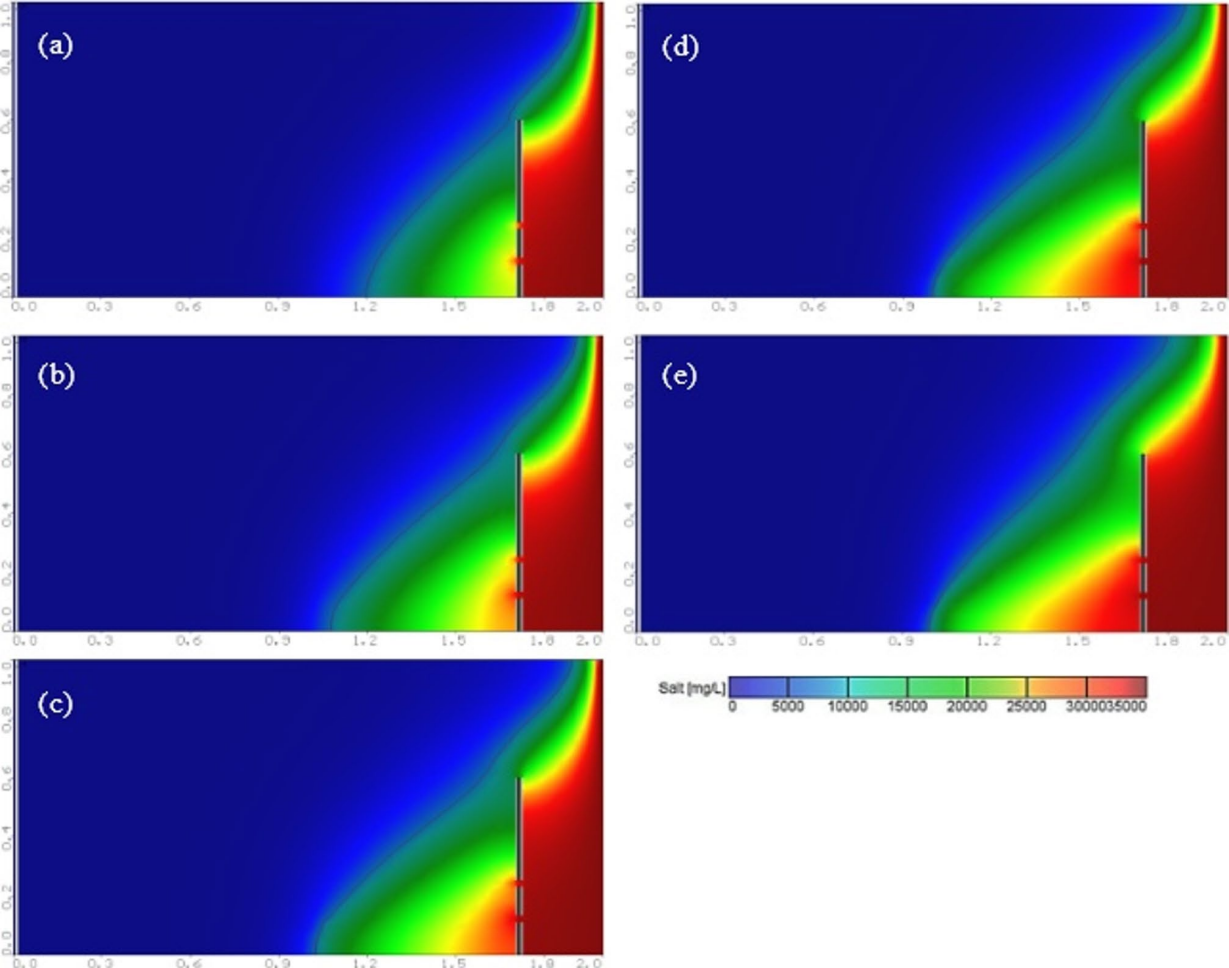
Effect of cracks and increased discharge on the interference of salt water with fresh water for different values of Q_w_/Q_f_: (a) 2%, (b) 4%, (c) 6%, (d) 8% and (e) 10%, dimension in meters, black line indicates 25% isohaline concentration.

Figure 7a shows the steady state saltwater interference wedge in the Henry problem after the installation of a underground dam with a height of H_d_=0.50 m to reduce seawater intrusion, at X_d_/L_o_=0.40, with a well location dimensionless ratio of X_w_/X_d_=1 and a well abstraction rate ratio of Q_w_=10%Q_f_. Figure 7b to h show a saltwater interference wedge in the case of a double-fractured underground dam with an upper fracture height of H_uf_=0.25 m and lower fracture height of H_lf_=0.12 m, at intervals of 5, 15, 25, 50, 75, 100 and 125 min. The diagram shows the significant progress of seawater interference within the reservoir, extending inland to a distance of 94.8 cm, and Fig. 8 shows the steady state saltwater interference wedge in the Henry seawater intrusion problem for the various values of an abstraction rate ratio of Q_w_=2%, 4%, 6%, 8% and 10% Q_f_.

#### Impact of underground dam location, well height and well location

**Fig. 9:**
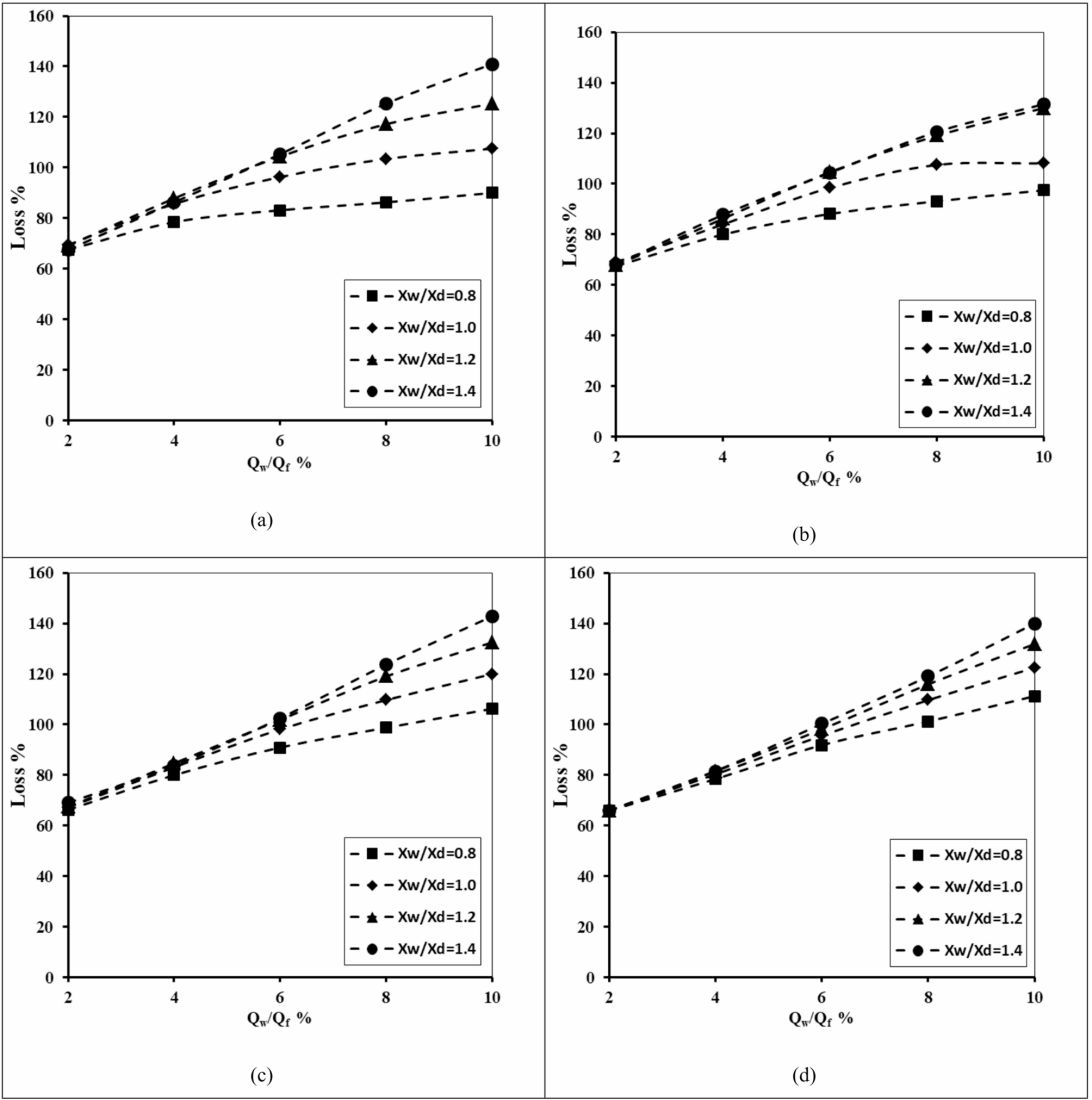
Relationship between the abstraction well rate and the losses of double-fractured underground dam efficiency for X_d_/L_o_ = 0.20, H_uf_=0.40 m, H_lf_=0.20 m, D_uf_=D_lf_=0.0075 m and for different X_w_/X_d_: (**a**) H_w_/H_d_ = 0.20, (**b**) H_w_/H_d_ = 0.40, (**c**) H_w_/H_d_ = 0.60 and (**d**) H_w_/H_d_ = 0.80.

Figure 9 describes the relation between the abstraction well rate ratio and the percentage of loss of efficiency of a double-fractured underground dam. This was done for X_d_/L_o_ = 0.20, H_uf_=0.40 m, H_lf_=0.20 m, D_uf_=D_lf_=0.0075 m and for various values of the ratio of well location (X_w_/X_d_) – 0.8, 1.0, 1.2, and 1.4. It can be confirmed from the figure that raising the well extraction rate led to a rise in the loss of efficiency ratio of the double-fractured underground dam; this is true for different values of the well depth ratio H_w_/H_d_. Expanding the value of Q_w_/Q_f_ led to a gradual increase in the loss percentage value. The loss percentage increased from 67.5% to 140.9%, 68.13% to 125.31%, 69% to 142.8% and from 65.9 to 140% for different values of the ratio of the height of the abstraction well (H_w_/H_d_) of 0.2, 0.4, 0.6 and 0.8, respectively. If the well is close to the underground dam and with a high well height ratio, the loss of efficiency was found to be high compared with the other cases. Raising the ratio of well location X_w_/X_d_ from 0.8 to 1.4 caused the loss of efficiency to rise for all tested values of abstraction rate ratios Q_w_/Q_f_ and well height ratio H_w_/H_d_.

**Table 4:** The losses of double-fractured underground dam efficiency for the underground dam distance X_d_/L_o_ = 0.20, for the minimum value of well height ratio H_w_/H_d_=0.2,and the maximum value of well height ratio H_w_/H_d_=0.8 and for different values of well location distances X_w_/X_d_.

Q_w_/Q_f_	Loss %							
	X_w_/X_d_=0.8		X_w_/X_d_=1.0		X_w_/X_d_=1.2		X_w_/X_d_=1.4	
	H_w_/H_d_		H_w_/H_d_		H_w_/H_d_		H_w_/H_d_	
	0.2	0.8	0.2	0.8	0.2	0.8	0.2	0.8
2	67.50	65.94	69.38	65.94	69.06	65.94	67.50	65.94
4	78.44	78.44	85.63	80.31	87.81	81.56	86.25	81.56
6	83.13	91.88	96.25	95.63	104.38	98.13	105.31	100.31
8	86.25	101.25	103.44	109.69	117.19	115.94	125.31	119.06
10	90.00	111.25	107.50	122.50	125.31	131.88	140.94	140.00

As presented in Table 4, the loss of efficiency percentage (Loss %) increased consistently with increasing the abstraction well rate ratio (Q_w_/Q_f_). At the minimum tested value (Q_w_/Q_f_=2%), the percentage of loss equals 67.5% for X_w_/X_d_=0.8 and H_w_/H_d_=0.2, and the same value was observed for X_w_/X_d_=1.4 and H_w_/H_d_=0.8. At the maximum tested value (Q_w_/Q_f_=10%), the percentage of loss ranged from 90% for X_w_/X_d_=0.8 and H_w_/H_d_=0.2 to 140% for X_w_/X_d_=1.4 and H_w_/H_d_=0.8. The impact of the abstraction well location ratio (X_w_/X_d_) was more noticeable than that of the abstraction well height ratio (H_w_/H_d_); for example, at an abstraction well rate ratio of Q_w_/Q_s_=10%, increasing the value of X_w_/X_d_ from 0.8 to 1.4 raised the losses by more than 50%, while increasing the value of H_w_/H_d_ from 0.2 to 0.8 typically raised losses by only 5–15%. These outcomes confirm that larger abstraction well distances produce higher efficiency losses; this was observed especially under greater abstraction rates.

**Fig. 10:**
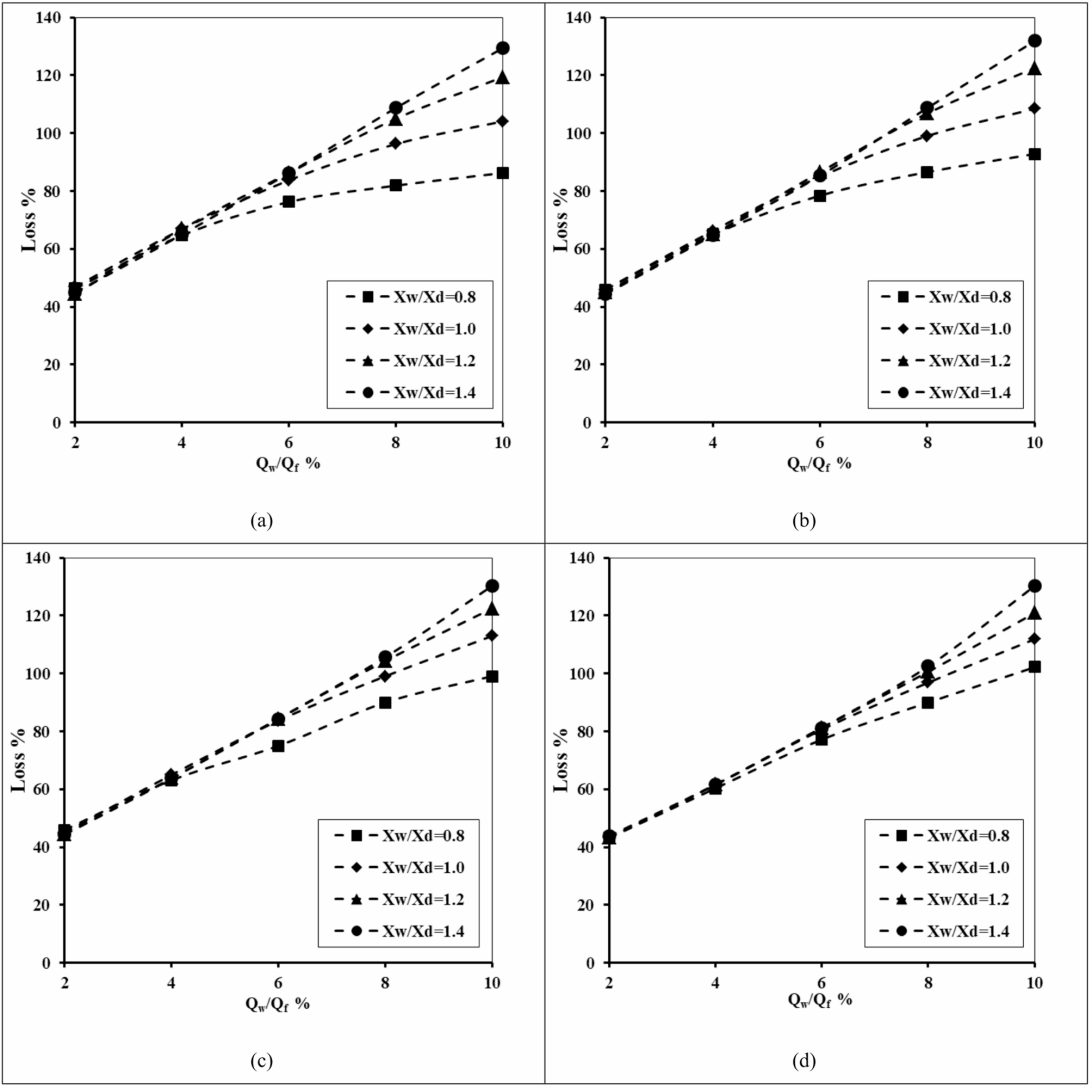
Relationship between the abstraction well rate and losses of double-fractured underground dam efficiency for X_d_/L_o_ = 0.40, H_uf_=0.40 m, H_lf_=0.20 m, D_uf_=D_lf_=0.0075 m and for different X_w_/X_d_ : (**a**) H_w_/H_d_ = 0.20, (**b**) H_w_/H_d_ = 0.40, (**c**) H_w_/H_d_ = 0.60 and (**d**) H_w_/H_d_ = 0.80.

The relationship between the abstraction well rate ratio and the double-fractured underground dam’s loss of efficacy is shown in Fig. 10. For X_d_/L_o_ = 0.40, H_uf_ = 0.40 m, H_lf_ = 0.20 m and D_uf_ = D_lf_ = 0.0075 m, this was carried out. For various well location ratio (X_w_/X_d_) values, these were 0.8, 1.0, 1.2 and 1.4. Increasing the location dam ratio X_d_/L_o_ from 0.2 to 0.4 caused a noticeable decrease in the loss of effectiveness values; this means that when the double-fractured underground dam is close to the saltwater boundary, the loss of effectiveness is expected to be high compared with the case where the dam existed far away from the seaside. The maximum value of the loss of effectiveness declined from 140.9% to 129.38%, 125.31% to 131.88%, 142.8% to 130.31% and from 140% to 129.38% when the underground dam location ratio increased from 0.2 to 0.4, for well height ratio H_w_/H_d_ equals 0.2, 0.4, 0.6 and 0.8 consequentially.

**Table 5:** The losses of double-fractured underground dam efficiency for the underground dam location ratio X_d_/L_o_ = 0.40, for the minimum value of well height ratio H_w_/H_d_=0.2, and the maximum value of well height ratio H_w_/H_d_=0.8 and for different values of the abstraction well location ratio X_w_/X_d_.

Q_w_/Q_f_	Loss %							
	X_w_/X_d_=0.8		X_w_/X_d_=1.0		X_w_/X_d_=1.2		X_w_/X_d_=1.4	
	H_w_/H_d_		H_w_/H_d_		H_w_/H_d_		H_w_/H_d_	
	0.2	0.8	0.2	0.8	0.2	0.8	0.2	0.8
2	46.25	43.13	46.88	43.13	44.38	43.13	45.00	43.75
4	64.69	60.31	66.88	61.56	66.88	61.56	65.00	61.56
6	76.25	77.19	83.75	80.63	86.25	81.25	86.25	81.25
8	81.88	90.00	96.25	96.88	105.00	100.63	108.75	102.50
10	86.25	102.19	104.06	111.88	119.38	120.94	129.38	130.31

As shown in Table 5, the percentage of loss of underground dam efficiency (Loss %) increased with the abstraction well rate ratio (Q_w_/Q_f_) across all abstraction well location ratios (X_w_/X_d_) and abstraction well height ratios (H_w_/H_d_). At the lowest value of the abstraction rate ratio (Q_w_/Q_f_=2%), the percentage of losses ranged from 43.1% for the well location ratio (X_w_/X_d_=0.8) and well height ratio (H_w_/H_d_=0.8) to 46.9% for (X_w_/X_d_=1.0) and (H_w_/H_d_=0.2). At the highest value of the abstraction rate ratio (Q_w_/Q_f_=10%), losses increased substantially, reaching values between 86.25% for (X_w_/X_d_=0.8, H_w_/H_d_=0.2) and 130.31% for (X_w_/X_d_=1.4, H_w_/H_d_=0.8). The outcomes confirm that increasing the abstraction well location ratio (X_w_/X_d_) has a significant impact on the efficiency loss than increasing the well height ratio (H_w_/H_d_). For example, at Q_w_/Q_f_=10%, increasing X_w_/X_d_ from 0.8 to 1.4 increased losses by 44%, while increasing H_w_/H_d_ from 0.2 to 0.8 typically increased the percentage of losses by 10–20%. These outcomes highlight the dominant role of abstraction well location in the efficiency loss of a double-fractured underground dam.

**Fig. 11:**
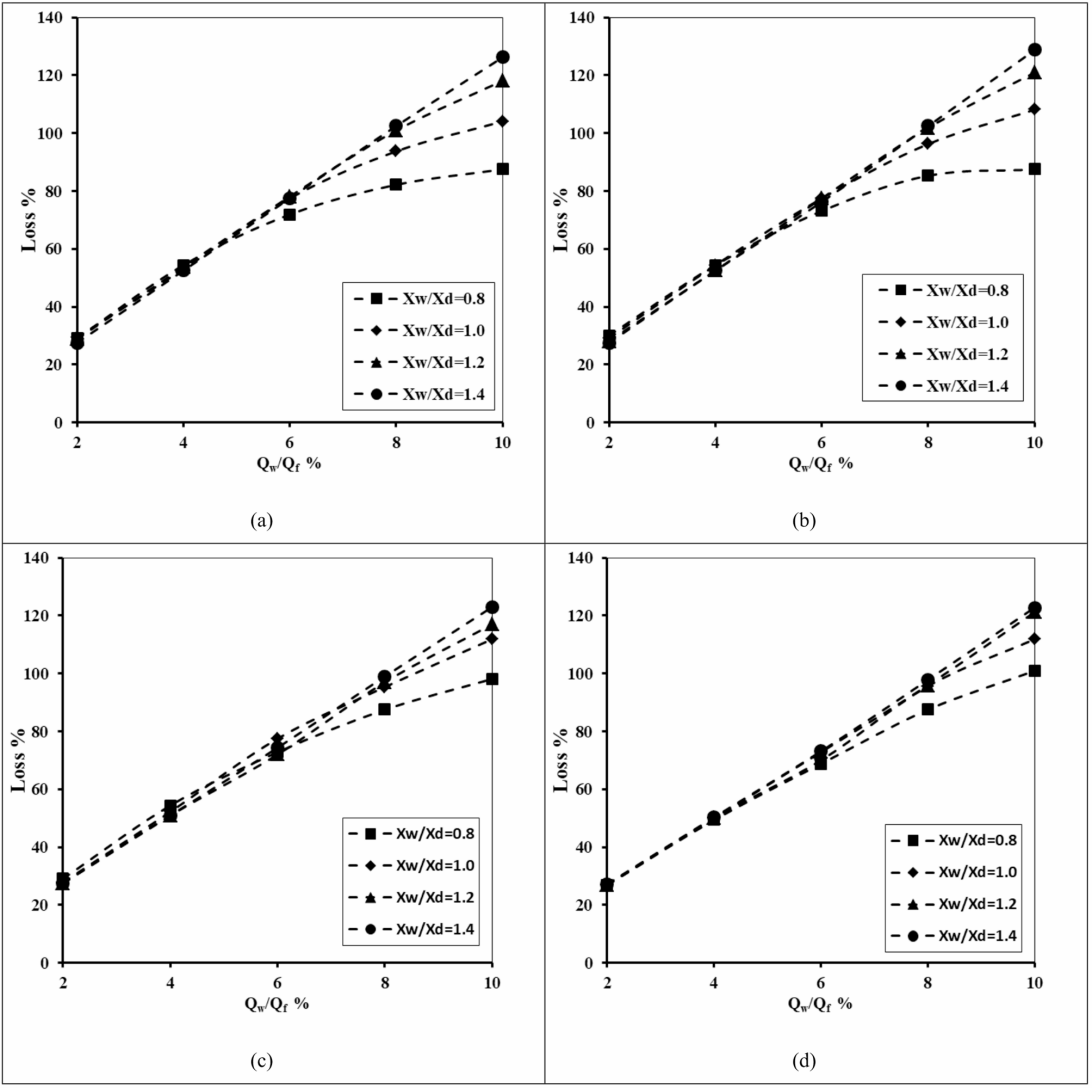
Relationship between the abstraction well rate and losses of double-fractured underground dam efficiency for X_d_/L_o_ = 0.60, H_uf_=0.40 m, H_lf_=0.20 m, D_uf_=D_lf_=0.0075 m and for different H_w_/H_d_ : (**a**) H_w_/H_d_ = 0.20, (**b**) H_w_/H_d_ = 0.40, (**c**) H_w_/H_d_ = 0.60 and (**d**) H_w_/H_d_ = 0.80.

Figure 11 illustrates the relationship between the abstraction well rate ratio and the double-fractured underground dam’s loss of efficacy. In addition to varying the well location ratio (X_w_/X_d_) values of 0.8, 1.0, 1.2 and 1.4, this was carried out for X_d_/L_o_ = 0.60, H_uf_ = 0.40 m, H_lf_ = 0.20 m and D_uf_ = D_lf_ = 0.0075 m. Increasing the ratio of underground dam (X_d_/L_o_) from 0.4 to 0.6 caused the loss of effectiveness of the underground dam to be minimised again compared with the previous two figures (Figs. 9 and 10). The difference in the loss of effectiveness has a slight increase with respect to increasing the well location ratio X_w_/X_d_ in the case of the abstraction rate ratio ranging from 2 to 6%, and the difference increases obviously when increasing the abstraction rate ratio from 6 to 10%. The difference of the loss of effectiveness ratio equals 14.25%, 19.81% and 17.91% for underground dam ratios X_d_/L_o_ equal to 0.2, 0.6 and 0.8, correspondingly.

**Table 6:** The losses of double-fractured underground dam efficiency for the underground dam location ratio X_d_/L_o_ = 0.60, for the minimum value of the well height ratio H_w_/H_d_=0.2 and the maximum value of well height ratio H_w_/H_d_=0.8 and for different values of the well location distance ratio X_w_/X_d_.

Q_w_/Q_f_	Loss %							
	X_w_/X_d_=0.8		X_w_/X_d_=1.0		X_w_/X_d_=1.2		X_w_/X_d_=1.4	
	H_w_/H_d_		H_w_/H_d_		H_w_/H_d_		H_w_/H_d_	
	0.2	0.8	0.2	0.8	0.2	0.8	0.2	0.8
2	29.00	26.88	29.38	27.00	28.75	26.88	27.50	27.19
4	54.38	49.38	53.00	50.00	53.75	50.00	52.50	50.31
6	71.88	68.75	77.50	70.00	78.13	72.81	77.50	73.13
8	82.19	87.50	93.75	95.63	100.94	95.94	102.50	97.81
10	87.50	100.94	104.06	111.88	118.13	121.25	126.25	122.81

As presented in Table 6, the values of efficiency loss increased gradually with the ratio of abstraction well rate (Q_w_/Q_f_); this is valid for all values of the abstraction well distance ratio (X_w_/X_d_) and the abstraction well height ratio (H_w_/H_d_). At the minimum tested abstraction rate (Q_w_/Q_f_=2%), the value of losses ranged narrowly between 26.88% and 29.38%, whereas at the maximum tested abstraction rate (Q_w_/Q_f_=10%), the losses increased noticeably, reaching values from 87.5% (X_w_/X_d_=0.8, H_w_/H_d_=0.2) up to 126.25% (X_w_/X_d_=1.4, H_w_/H_d_=0.2). The impact of the well location ratio X_w_/X_d_ was again more pronounced than the well height ratio H_w_/H_d_. For example, at the maximum abstraction rate of Q_w_/Q_f_=10%, increasing X_w_/X_d_ from 0.8 to 1.4 raised the loss values by approximately 39%, whereas increasing the value of H_w_/H_d_ from 0.2 to 0.8 typically raised losses by 10–15%. These findings confirm that greater abstraction well distances significantly intensify losses, predominantly under higher values of well abstraction rates.

**Fig. 12:**
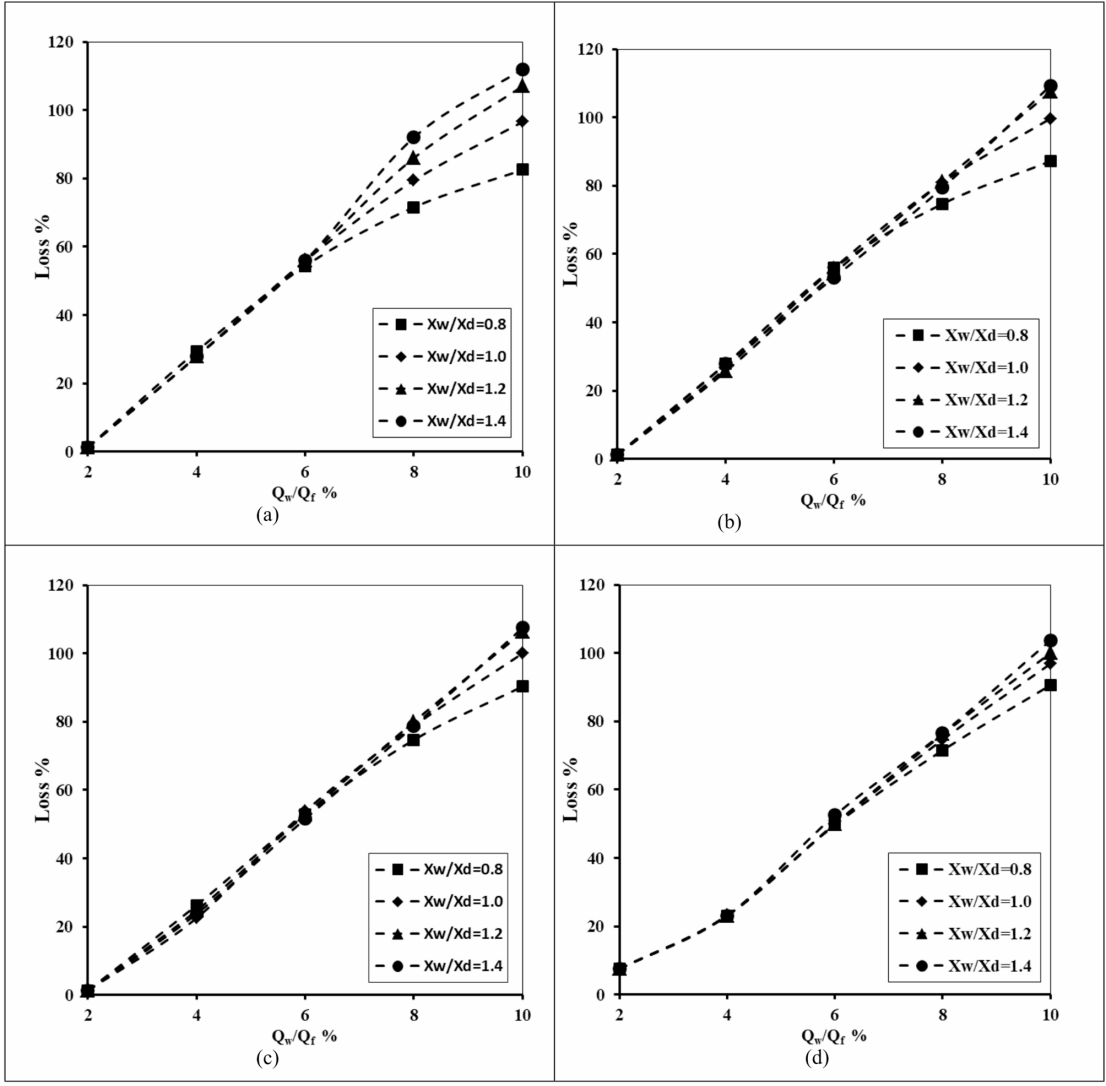
The abstraction well rate relationship to loss of double-fractured underground dam effectiveness for X_d_/L_o_ = 0.80, H_uf_=0.40 m, H_lf_=0.20 m, D_uf_=D_lf_=0.0075 m and for different X_w_/X_d_ : (**a**) H_w_/H_d_ = 0.20, (**b**) H_w_/H_d_ = 0.40, (**c**) H_w_/H_d_ = 0.60 and (**d**) H_w_/H_d_ = 0.80.

Figure 12 shows how the abstraction well rate ratio and the double-fractured underground dam’s loss of effectiveness are related. This was done for the following values: X_d_/L_o_=0.20, H_uf_=0.40 m, H_lf_ =0.20 m and D_uf_=D_lf_ =0.0075 m. The well location ratio (X_w_/X_d_) was varied between 0.8, 1.0, 1.2 and 1.4. When the underground dam’s X_d_/L_o_ ratio was increased to 0.8, its effectiveness decreased once more in comparison to 0.2, 0.4 and 0.6. Positioning the underground dam away from the seawater boundary at X_d_/L_o_ equals 0.8 has a loss of effectiveness noticeably small compared to 0.2, 0.4 and 0.6. The value of the loss of effectiveness boosted from 1.25 to 112%, 1.25 to 109.38%, 1.25 to 107.5% and from 7.5 to 103.5% for height well ratio H_w_/H_d_ equals 0.2, 0.4, 0.6, and 0.8 consequentially. Increasing the underground dam location ratio X_d_/L_o_ from 0.6 to 0.8 led to noticeable decrease of the loss of effectiveness ratio; this difference equals 14.25, 19.37, 15.5 and 19.31 for a well height ratio equal to 0.2, 0.4, 0.6, and 0.8 correspondingly.

**Table 7:** The loss of double-fractured underground dam efficiency for underground dam distance ratio of X_d_/L_o_ = 0.80, for the minimum value of well height ratio H_w_/H_d_=0.2 and the maximum value of well height ratio H_w_/H_d_=0.8 and for different values of abstraction well distance ratio X_w_/X_d_.

Q_w_/Q_f_	Loss %							
	X_w_/X_d_=0.8		X_w_/X_d_=1.0		X_w_/X_d_=1.2		X_w_/X_d_=1.4	
	H_w_/H_d_		H_w_/H_d_		H_w_/H_d_		H_w_/H_d_	
	0.2	0.8	0.2	0.8	0.2	0.8	0.2	0.8
2	1.25	7.50	1.25	7.50	1.25	7.50	1.25	7.50
4	29.38	23.13	27.81	23.13	27.81	23.13	27.81	23.13
6	54.38	49.69	55.94	50.00	55.94	50.00	55.94	52.50
8	71.56	71.56	79.38	74.69	86.00	76.25	92.00	76.56
10	82.50	90.63	96.56	96.88	107.00	100.00	112.00	103.75

Table 7 presents the variation in the double-fractured underground dam efficiency losses in regard to the well abstraction rate ratio (Q_w_/Q_f_) at various underground dam distances ratio X_d_/L_o_=0.8. At the lowest abstraction rate (Q_w_/Q_f_=2%), the percentage of losses were minimal, ranging only from 1.25% to 7.5% depending on the well height ratio H_w_/H_d_ and the abstraction well ratio X_w_/X_d_. On the other hand, as the well abstraction rate increased, the losses rose sharply. For example, at the maximum abstraction well ratio of Q_w_/Q_f_=10%, loss values extended from 82.50% (X_w_/X_d_=0.8, H_w_/H_d_=0.2) to 112% (X_w_/X_d_=1.4, H_w_/H_d_=0.2). The results confirm that increasing the abstraction well distance ratio (X_w_/X_d_) consistently produces higher efficiency losses, whereas the impact of the abstraction well height ratio (H_w_/H_d_) is relatively moderate. This pattern confirms that the location of the abstraction well exerts the dominant control on the loss variation, particularly under higher pumping conditions.

#### Impact of underground dam depth

**Fig. 13:**
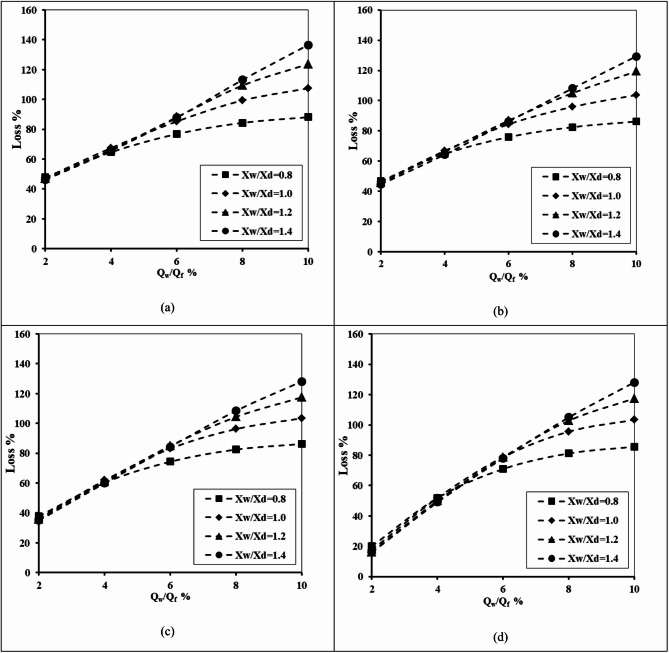
The abstraction well rate relationship to the loss of double-fractured underground dam efficiency for H_uf_=0.40 m, H_lf_=0.20 m, D_uf_=D_lf_=0.0075 m, for different tested values of X_w_/X_d_ and for diverse values of underground dam depth: (**a**) H_d_ = 0.50, (**b**) H_d_ = 0.60, (**c**) H_d_ = 0.70 and (**d**) H_d_ = 0.80.

For H_uf_=0.40 m, H_lf_=0.20 m and D_uf_=D_lf_=0.0075 m, Fig. 13 shows the relationship between the well abstraction discharge and loss of double-fractured underground dam efficiency for various tested values of X_w_/X_d_ and different values of underground dam depth: H_d_ = 0.50, 0.60, 0.70, and 0.80 m. The outcomes of this figure confirmed that the values of the loss of efficiency are quite high in the case of short underground dam depth compared with a higher dam depth. The loss of efficiency increased gradually for different tested abstraction rates and various values of underground dam depths. In the case of a shorter underground dam, the seawater can move above the top of the dam, especially in cases where the screen of the well is close and near the underground dam. The loss percentage increased from 46.25 to 136.56%, 44.69 to 129.38%, 35 to 128.13% and from 15.94 to 128.13% for an underground dam depth H_d_ equal to 0.5, 0.6, 0.7, and 0.8 m. Positioning the groundwater well closer to the double-fractured underground dam achieved higher values of loss of effectiveness for a dam in mitigating the SWI into the aquifer. The loss of effectiveness rose by the following percentages: 48.43, 43.13, 41.88 and 40.15% by moving the abstraction well from X_w_/X_d_=0.8 to 1.4, for H_d_ equals 0.5, 0.6, 0.7 and 0.8 m.

**Table 8:** The losses of double-fractured underground dam efficiency for underground dam distance ratio X_d_/L_o_ = 0.80, for the minimum value of well height ratio H_w_/H_d_=0.2, the maximum value of well height ratio H_w_/H_d_=0.8 and for different values of X_w_/X_d_.

Q_w_/Q_f_	Loss %							
	H_d_=0.5		H_d_=0.6		H_d_=0.7		H_d_=0.8	
	X_w_/X_d_		X_w_/X_d_		X_w_/X_d_		X_w_/X_d_	
	0.8	1.4	0.8	1.4	0.8	1.4	0.8	1.4
2	47.81	46.25	46.88	44.69	37.81	35.00	20.00	15.94
4	64.69	65.63	64.69	64.38	60.31	60.00	51.88	49.06
6	76.88	87.81	75.94	86.25	74.38	84.38	70.94	78.13
8	84.38	113.13	82.50	108.13	82.50	108.44	81.25	105.00
10	88.13	136.56	86.25	129.38	86.25	128.13	85.63	128.13

Table 8 displays the impact of well abstraction rate (Q_w_/Q_f_), abstraction well height ratio (H_w_/H_d_) and well location ratio (X_w_/X_d_) on the underground dam efficiency losses at various well location ratios (X_w_/X_d_) 0.8 and 1.4. At the lowest abstraction rate ratio (Q_w_/Q_f_=2%), losses were relatively modest, ranging from 15.9% (H_w_/H_d_=0.8, X_w_/X_d_=1.4) to 47.81% (H_w_/H_d_=0.5, X_w_/X_d_=1.4). Increasing the abstraction rate ratio led to increase the losses substantially. For instance, at maximum abstraction rate ratio (Q_w_/Q_f_=10%), the values extended from 85.63% (H_w_/H_d_=0.7, X_w_/X_d_=0.8) up to 136.56% (H_w_/H_d_=0.5, X_w_/X_d_=1.4). The findings confirm that an abstraction well positioned closer to a fractured underground dam (lower values of X_w_/X_d_) and with smaller well height ratios (H_w_/H_d_) are more prone to efficiency losses. In addition, the variation among the abstraction well height (H_w_/H_d_) values confirms that the well height ratio employs a secondary but still significant, especially for varied abstraction rates.

#### Impact of upper fracture height and upper fracture diameter

**Fig. 14:**
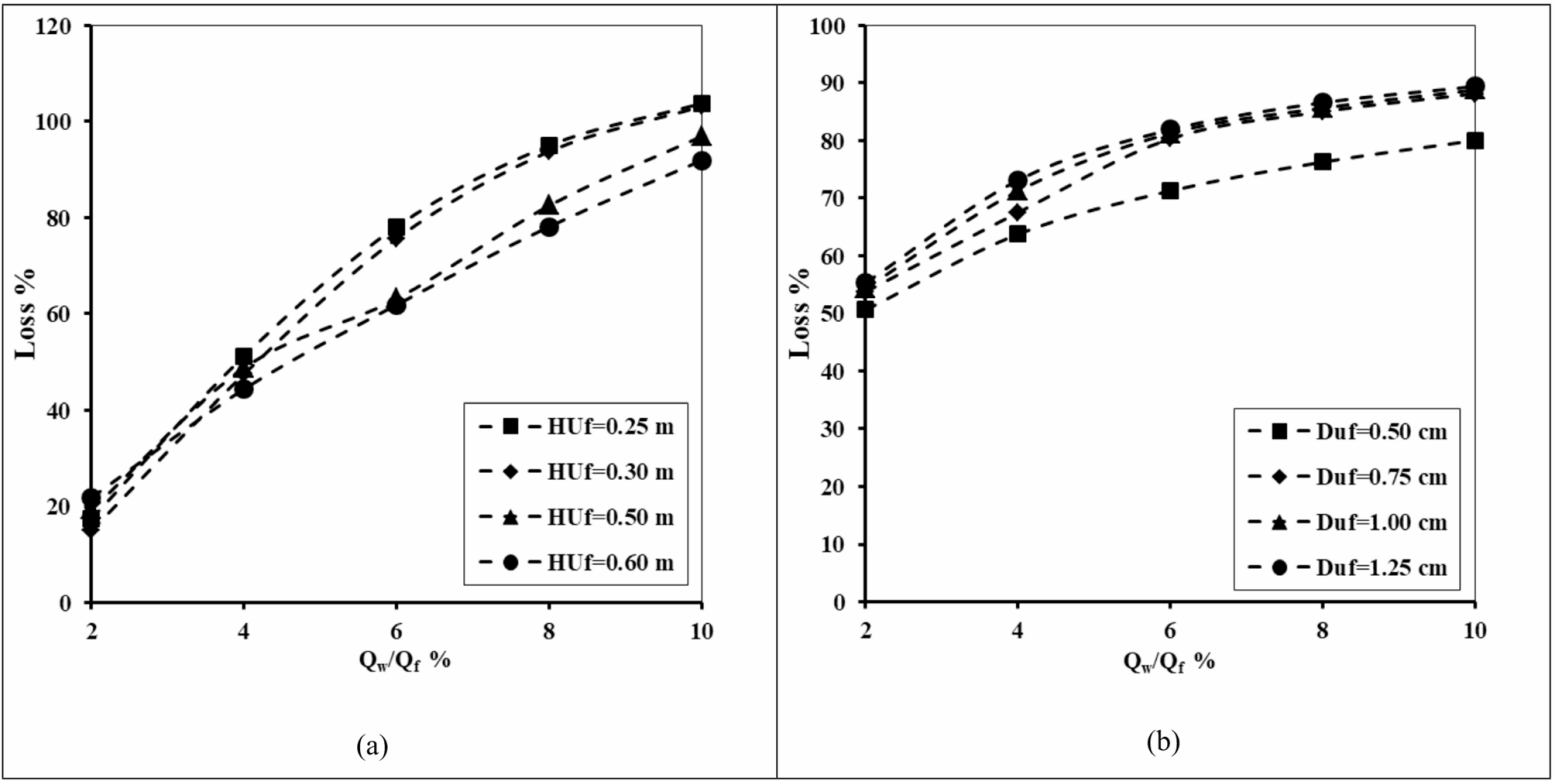
The abstraction well rate relation to the loss of double-fractured underground dam efficiency for X_d_/L_o_ = 0.40, H_d_=0.8 m, H_w_/H_d_=0.2, H_lf_= 0.2 m, D_lf_ =0.0075 m, X_w_/X_d_=1.0 and for different values of: (**a**) Height of upper fracture (H_uf_) and (**b**) Diameter of upper fracture (D_uf_).

Figure 14 shows the relationship between the well abstraction rate and loss of the double-fractured underground dam efficiency for X_d_/L_o_=0.40, H_d_=0.8 m, H_w_/H_d_=0.2, H_lf_=0.2 m, D_lf_ =0.0075 m, X_w_/X_d_=1.0 and for different values of height of the upper fracture (H_uf_), and diameter of the upper fracture (D_uf_). Regarding the impact of variations in the upper fracture height on the losses of effectiveness of the double-fractured underground dam, a study was conducted for different values of (H_uf_) of 0.25, 0.30, 0.50 and 0.60 m. Increasing the ratio of abstraction rate from 2% to 10% caused the loss of double-fractured underground dam to increase from 17.75 to 103.75% for the height of the upper fracture equal to 0.25. Furthermore, the loss value of the double-fracture efficiency increased from 55.31 to 89.38% for the diameter of the upper fracture equal to 1.25 cm. Increasing the rate of abstraction causes the loss of effectiveness to increase with respect to different tested values of upper fracture height. Increasing the height of the upper fracture led to a decline in the value of the loss percentage. In the case of a double fracture close to the base of the underground dam, a high volume of saline water with high density passes through the opening of the fractures in the underground dam to be upstream of the underground dam. As a result, the length of SWI wedges length increased, and the dam loses a high value of its efficiency. In addition, it seems that the diameter of the fracture also affects the loss of efficiency of the underground dam. Expanding the dimension of the opening through the underground dam causes an increase in the volume of saline water passing through it; in addition, the efficiency decreases due to the increase in the seawater wedge length.

**Table 9:** The losses of double-fractured underground dam efficiency for the underground dam location ratio X_d_/L_o_ = 0.40, for various abstraction well ratios Q_w_/Q_f_, for different values of upper fracture height H_uf_ and for different values of upper fracture diameter D_uf_.

Q_w_/Q_f_	Loss %							
	H_uf_				D_uf_			
	0.25	0.30	0.50	0.60	0.25	0.30	0.50	0.60
2	15.00	17.50	19.38	21.88	50.63	53.75	54.38	55.31
4	47.50	51.25	48.75	44.38	63.75	67.50	71.25	73.13
6	75.63	78.13	63.13	61.88	71.25	80.31	81.25	81.88
8	93.75	95.00	82.50	78.13	76.25	85.00	85.63	86.56
10	103.13	103.75	96.88	91.88	80.00	88.13	88.75	89.38

Table 9 highlights the impact of the upper fracture height (H_uf_) and the upper fracture diameter (D_uf_) on the underground dam efficiency losses for various well abstraction rates (Q_w_/Q_f_). For the minimum abstraction well rate ratio Q_w_/Q_f_=2%, losses associated with (H_uf_) ranged from 15% for H_uf_=0.25 to 21.88% for H_uf_=0.60, whereas losses values related to the diameter of the upper fracture varied between 50.63% (D_uf_=0.25) and 55.31% (D_uf_=0.6). As the abstraction well rate rose, losses increased considerably. At the maximum tested abstraction rate ratio of Q_w_/Q_f_=10%, losses extended to 103.13% for H_uf_=0.25 and 91.88% for H_uf_=0.6, while D_uf_ produced even lower losses values, varying between 80% (D_uf_=0.25) and 89.4% (D_uf_=0.6). The outcomes reveal that increasing the upper fracture height H_uf_ tends to lower the efficiency losses, especially at higher abstraction well rates. In addition, larger values of upper fracture diameter are consistently associated with higher losses. This shows the fracture geometry, especially the diameter of the upper fracture, has a significant impact on the underground dam efficiency in controlling SWI.

#### Impact of lower fracture height and lower fracture diameter

**Fig. 15:**
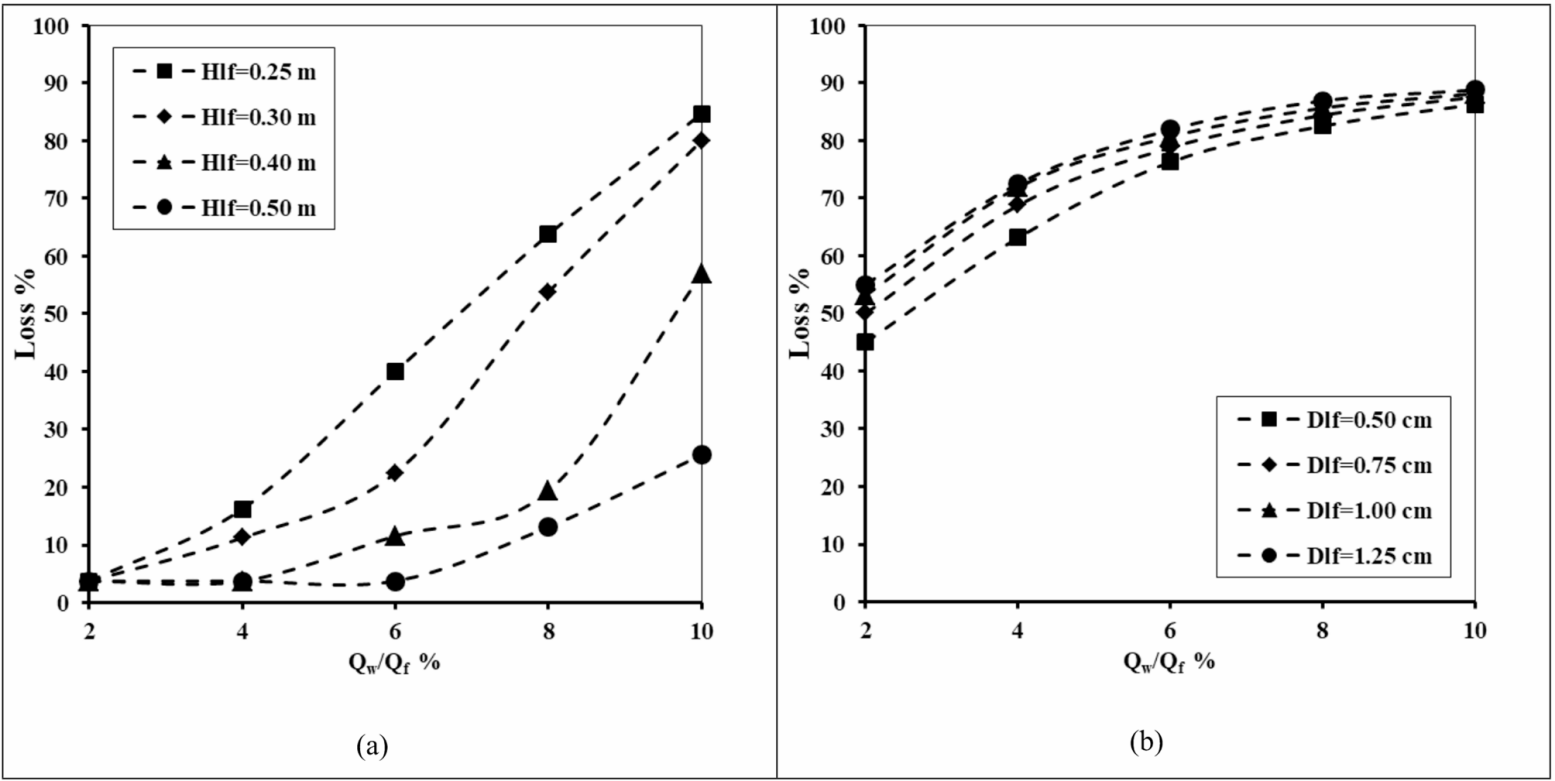
The abstraction well rate relation with the loss of double-fractured underground dam efficiency for X_d_/L_o_ = 0.40, H_d_=0.8 m, H_w_/H_d_=0.2, H_uf_= 0.6 m, D_uf_ =0.0075 m, X_w_/X_d_=1.0 and for different values of: (**a**) Height of lower fracture (H_uf_) and (**b**) Diameter of lower fracture (D_lf_).

Figure 15 illustrates the relationship between the abstraction well discharge and failure of the double-fractured underground dam’s effectiveness for X_d_/L_o_=0.40, H_d_=0.8 m, H_w_/H_d_=0.2, H_lf_=0.2 m, D_lf_ =0.0075 m, X_w_/X_d_=1.0 and for varying values of height of the lower fracture (H_lf_) and diameter of the lower fracture (D_lf_). In order to determine how variations in the upper fracture height affected the double-fractured underground dam’s loss of effectiveness, tests were conducted for a range of (H_lf_) values, including 0.25, 0.30, 0.50 and 0.60 m. The loss of the double-fractured underground dam’s efficiency increased dramatically, from 3.75 to 84.69% when the rate of abstraction ratio was increased from 2% to 10%, with the height of the lower fracture equal to 0.25. Furthermore, when the higher fracture’s diameter was 1.25 cm, the dam’s efficiency’s loss value rose gradually from 55.00 to 88.75%. With regard to various evaluated values of lower fracture height, the loss of effectiveness increases as the rate of abstraction increases. The value of the loss percentage decreased as the height of the top fracture increased. When there is a double fracture near the bottom of the underground dam, a large amount of dense, salty water flows upstream through the openings in the dam. As a result, the saltwater wedges’ length increased, and the dam’s effectiveness dropped significantly. Furthermore, it appears that the underground dam’s loss of efficiency is also influenced by the fracture’s diameter. The volume of saltwater flowing through the underground dam grows as the opening’s lower diameter increases, and the efficiency also declines as the seawater wedge length increases.

**Table 10:** The losses of double-fractured underground dam efficiency for the underground dam location ratio X_d_/L_o_ = 0.40, for various abstraction well ratio Q_w_/Q_f_, for different values of lower fracture height H_lf_ and for different values lower fracture diameter D_lf_.

Q_w_/Q_f_	Loss %							
	H_lf_				D_lf_			
	0.25	0.30	0.50	0.60	0.25	0.30	0.50	0.60
2	3.75	3.75	3.75	3.75	45.00	50.00	53.13	55.00
4	16.25	11.25	3.75	3.75	63.13	68.75	71.88	72.50
6	40.00	22.50	11.56	3.75	76.25	78.75	80.63	81.88
8	63.75	53.75	19.38	13.13	82.50	84.38	85.63	86.88
10	84.69	80.00	56.88	25.63	86.25	87.50	88.13	88.75

Table 10 displays the impact of lower fracture height (H_lf_) and lower fracture diameter (D_lf_) on the loss efficiency of the underground dam under different abstraction rates (Q_w_/Q_f_). For the height of the lower fracture (H_lf_), the findings show a clear declining trend in losses as the height of the lower fracture increases. At a higher value of the well abstraction rate Q_w_/Q_f_=10%, losses declined from 84.7% (H_lf_=0.25) to only 25.6% (H_lf_=0.60), showing that a higher elevation of the lower fracture noticeably helps reduce efficiency loss. On the other hand, the lower fracture diameter (D_lf_) exhibited the reverse behaviour. For the same case of abstraction well rate, the efficiency losses increased gradually from 86.3% with D_lf_=0.25 and reached 88.8% with D_lf_=0.6, indicating that a larger lower fracture aperture is linked with greater inefficiency. These results confirm that the geometry of the lower fracture strongly affects the underground dam efficiency to control SWI, whereas increasing the elevation of the lower fracture (H_lf_) can minimise the losses, and enlarging the lower fracture diameter (D_lf_) exhibited the reverse behaviour.

#### Impact of seawater density

**Fig. 16:**
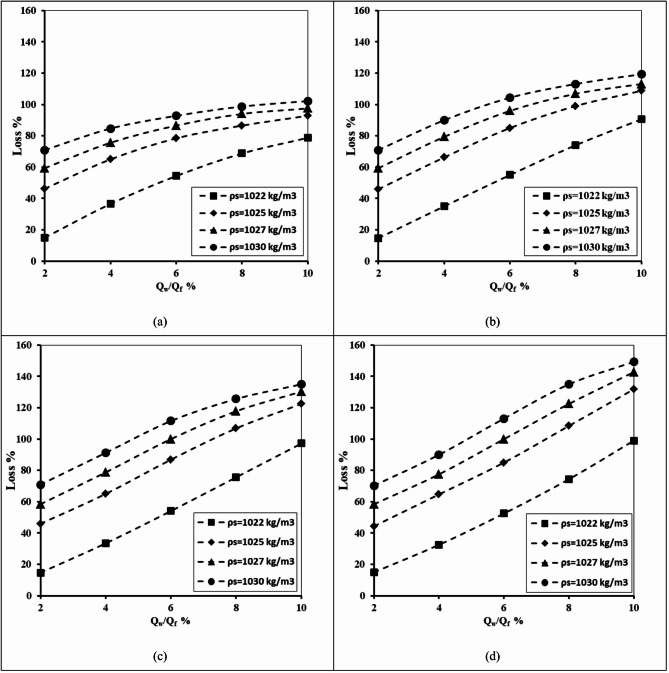
The abstraction well rate relation with the loss of double-fractured underground dam efficiency for X_d_/L_o_ = 0.40, H_w_/H_d_=0.4, H_uf_=0.40 m, H_lf_=0.20 m, D_uf_=D_lf_= 0.0075 m and for diverse values of seawater density and for different locations of well: (**a**) X_w_/X_d_=0.8, and (**b**) X_w_/X_d_=1.0, (**c**) X_w_/X_d_=1.2 and (**d**) X_w_/X_d_=1.4.

The relationship between the abstraction well discharge and the losses in the double-fractured underground dam effectiveness is depicted in Fig. 16 for the following varied seawater density values and well locations X_w_/X_d_=0.8, 1.0, 1.2 and 1.4. It can be confirmed from the figures that the value of the loss efficiency rose gradually for all tested seawater densities with the increase of the well abstraction rate from 2 to 10%. By increasing the well location distance ratio from 0.8 to 1.4, the values of percentage loss increased for all tested values of saltwater density. Raising the value of seawater density from 1022 to 1030 kg/m^3^ led to an upsurge in the length of the seawater intrusion wedge, and the loss of efficiency of the double-fractured underground dam increased. For a seawater density of 1022 kg/m^3^, the loss of efficiency increased by 63.75, 75.63, 82.19 and 78.44% respectively; in addition the loss percentage value rose by 31.25, 48.44, 64.06 and 78.55% consequentially for (X_w_/X_d_) equals 0.8, 1.2, 1.2 and 1.4.

**Table 11:** The loss of double-fractured underground dam efficiency for underground dam distance ratio X_d_/L_o_ = 0.80, for various values of saltwater density (ρ_f_) and for various values of well location ratio X_w_/X_d_.

Q_w_/Q_f_	Loss %							
	ρ_f_ = 1022		ρ_f_ = 1025		ρ_f_ = 1027		ρ_f_ = 1030	
	X_w_/X_d_		X_w_/X_d_		X_w_/X_d_		X_w_/X_d_	
	0.8	1.4	0.8	1.4	0.8	1.4	0.8	1.4
2	15.00	15.00	46.25	44.38	59.38	58.44	70.94	70.31
4	36.56	32.50	65.00	64.69	75.63	77.50	84.69	90.00
6	54.38	52.50	78.44	85.00	86.56	100.00	92.81	113.13
8	68.75	74.38	86.56	108.44	94.00	122.50	98.75	135.00
10	78.75	99.06	92.81	131.88	97.50	142.81	102.19	149.38

Table 11 demonstrates the influence of seawater density (ρ_s_) on the efficiency losses of the fractured underground dam under various well abstraction rates (Q_w_/Q_f_) and abstraction well distance ratios (X_w_/X_d_). At the minimum abstraction rate (Q_w_/Q_f_=2%), losses were relatively minor, ranging from 15% (ρ_s_ = 1022, X_w_/X_d_=0.8) to 149.38% (ρ_s_ = 1030, X_w_/X_d_=1.4). However, with an increase in the abstraction rate ratio Q_w_/Q_f_, losses rose significantly. For example, at Q_w_/Q_f_=10%, efficiency losses varied from 78.75% (ρ_s_ = 1025, X_w_/X_d_=0.8) to 149.38% (ρ_s_ = 1030, X_w_/X_d_=1.4). The outcomes confirmed that higher seawater density constantly led to higher efficiency losses. In addition, larger abstraction well location ratios (X_w_/X_d_) magnify this impact. These outcomes highlight that seawater salinity, combined with abstraction well location, plays a significant role in controlling fractured underground dam efficiency under various abstraction rates.

#### Comparison between a single- and double-fractured underground dam

**Fig. 17:**
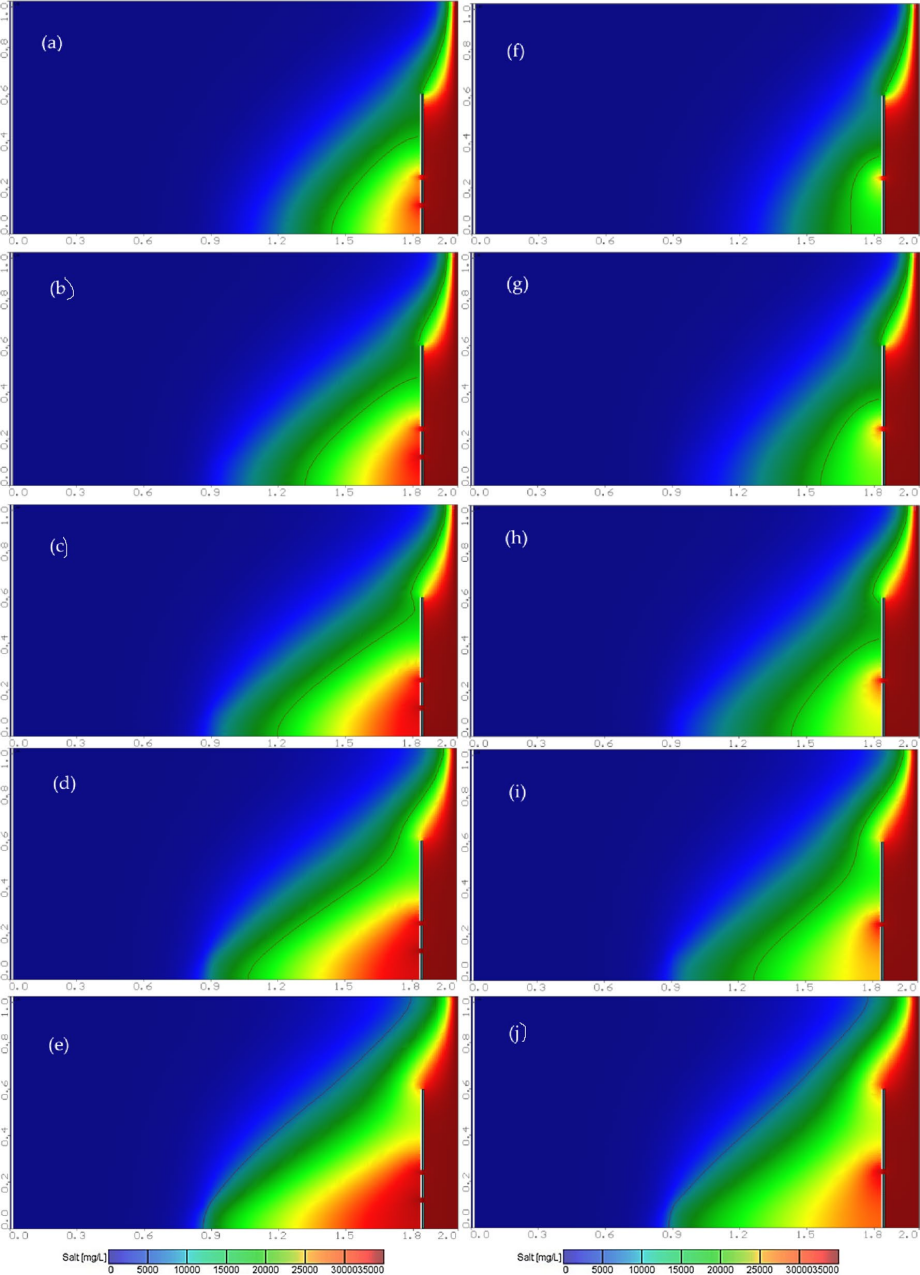
Evolution of the steady-state interface between salt and fresh water for double-fractured underground dam for various abstraction well ratios of Q_w_/Q_f_: (**a**) 2%, (**b**) 4%, (**c**) 6%, (**d**) 8% and (**e**) 10%, and for a single-fractured dam for various abstraction well ratios of Q_w_/Q_f_: (**f**) 2%, (**g**) 4%, (**h**) 6%, (**i**) 8% and (**j**) 10%, dimension in meters, and black line indicates 25% isohaline concentration.

**Fig. 18:**
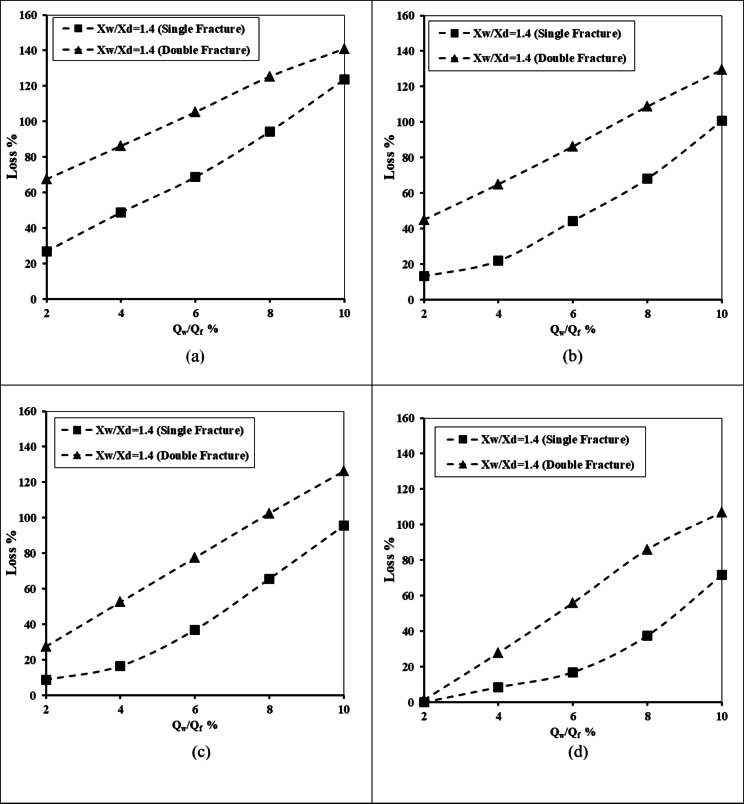
The abstraction well rate relation with the loss of single- and double-fractured underground dam efficiency for X_d_/L_o_ = 0.20, H_w_/H_d_=0.2, for a single fracture H_uf_=0.40 m, for double-fracture H_uf_=0.40 m H_lf_=0.20 m, D_uf_=D_lf_= 0.0075 m, for X_w_/X_d_=1.4, for different underground dam distance ratios X_d_/L_o_: (**a**) 2%, (**b**) 4%, (**c**) 6%, and (**d**) 8%.

Figure 17 shows the steady-state seawater-freshwater interface for a double-fractured underground dam for various abstraction well ratios Q_w_/Q_f_: (a) 2%, (b) 4%, (c) 6%, (d) 8% (e) 10%, and for single-fractured underground dam for various abstraction well ratios Q_w_/Q_f_: (f) 2%, (g) 4%, (h) 6%, (i) 8% and (j) 10%. Figure 18 presents the abstraction well rate relation to the loss of single- and double-fractured underground dam effectiveness for X_d_/L_o_ = 0.20, H_w_/H_d_=0.2. For the single-fractured dam H_uf_ equals 0.40 m, for the double-fractured dam H_uf_=0.40 m, and H_lf_=0.20 m, D_uf_=D_lf_= 0.0075 m, for the well location ratio of X_w_/X_d_=1.4; for different underground dam distances ratio equals 2%, 4%, 6%, 8% and 10%.

The results of Figs. 17 and 18 confirm that the double-fractured underground dam lost a high percentage of its efficiency in mitigation of seawater intrusion compared with the single-fractured underground dam. The existence of the two fractures contributes to the passing of a high volume of saline water compared with the single fracture; as a result of that, the concentration line of 17,500 mg/L intruded more inland into the aquifer in the case of double-fractured compared with the single-fractured. This is valid for various abstraction rate ratios of 2%, 4%, 6%, 8% and 10%. In addition, increasing the abstraction rate ratio causes the underground dam to lose a high value of its efficiency, because increasing the rate led to an increase of the intrusion length, and the area of the seawater wedge and the repulsion ratio of the saltwater wedge declined.

**Table 12:** The loss of single and double-fractured underground dam efficiency for various underground dam location ratios X_d_/L_o_ = 0.80.

Q_w_/Q_f_	Loss %							
	Single Fracture				Double Fracture			
	X_d_/L_o_				X_d_/L_o_			
	0.2	0.4	0.6	0.8	0.2	0.4	0.6	0.8
2	26.88	13.13	8.75	0.00	67.50	45.00	27.50	1.25
4	48.75	21.88	16.56	8.44	86.25	65.00	52.50	27.81
6	68.75	44.38	36.88	16.88	105.31	86.25	77.50	55.94
8	94.38	68.13	65.63	37.50	125.31	108.75	102.50	86.00
10	123.75	100.63	95.63	71.56	140.94	129.38	126.25	107.00

Table 12 compares the variation of the efficiency losses of single- and double-fractured underground dams at various location ratios (X_d_/L_o_) and various abstraction rates (Q_w_/Q_f_). For single-fractured underground dam systems, the loss rose steadily with abstraction rate, varying from 0.0% at Q_w_/Q_f_=2% and X_d_/L_o_=0.8 to a maximum of 123.75% at Q_w_/Q_f_=10% and X_d_/L_o_=0.2. On the other hand, double-fractured underground dam systems exhibited consistently higher losses under the same conditions, with values rising from 1.25% (Q_w_/Q_f_=2% and X_d_/L_o_=0.8) to 140.90% (Q_w_/Q_f_=10% and X_d_/L_o_=0.2). The comparison between single- and double- fractured systems confirms that the existence of a double-fracture increases the efficiency losses through all abstraction rates and abstraction well locations. Furthermore, in the both fracture cases, higher underground dam location ratio X_d_/L_o_ values were connected with declined losses values, but the relative improvement was more pronounced in single-fracture dams. These outcomes confirm that while double-fractured systems tend to experience greater losses overall, optimising the abstraction well location ratio significantly mitigates their negative influence.

Laabidi et al. ^38^ investigated the influence of fractures in concrete cutoff walls on the dynamics of SWI. Different fracture configurations were tested, and it was confirmed that fractured walls lose up to 63% of their effectiveness in SWI control. Sensitivity analysis showed that fracture aperture, well location, fracture position and seawater density significantly influence the loss of effectiveness. Armanuos et al. ^16^ examined fractured subsurface dams under the influence of groundwater extraction using SEAWAT modelling. They investigated variables such as dam height and location, well depth and placement, abstraction rate, fracture size and seawater density. Their results confirmed that higher groundwater extraction rates significantly reduce the dam’s effectiveness, with losses varying from about 35% to 93% depending on the abstraction intensity. The well location ratio also has a significant impact; positioning wells far away increased the seawater movement though the dam fractures. Overall, the impact of fractures combines with excessive groundwater abstraction intensity of SWI and reduces the underground dam efficiency.

Both studies revealed that fractures in concrete underground dams significantly affect their performance against SWI. Whereas Laabidi et al. ^38^ highlight the importance of fracture geometry and dam location to determine the flow pathways, Armanuos et al. ^16^ extend this by incorporating groundwater abstraction, presenting how abstraction rate accelerates the decline of the fractured underground dam’s efficiency. Together, both studies highlight that structural integrity and abstraction management are crucial for the long-term success of underground barriers built to protect freshwater in coastal aquifers. The current study concluded that the presence of two cracks in an underground dam, along with the presence of well withdrawals, reduces the dam’s efficiency in controlling SWI. The percentage of efficiency losses in the case of a double-fractured underground dam ranged from 27.5 to 140%, compared with 14.7 to 63% in a single-fractured dam without an abstraction well and from 8.75 to 123% for a single-fractured dam combined with an abstraction well, under the same conditions. The losses depend mainly on the fracture aperture and location, dam height and location and the abstraction rate.

### Real case study (the Akrotiri coastal Aquifer, Cyprus)

**Fig. 19:**
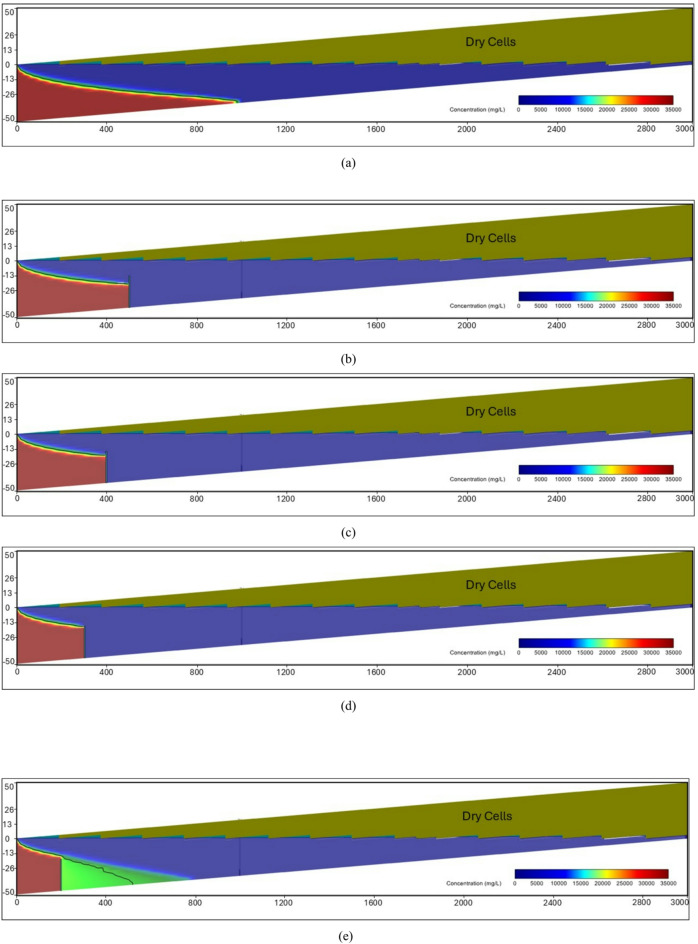
Steady-state seawater intrusion distribution for the Akrotiri coastal aquifer: (**a**) without underground dam, and after the embedment of the underground dam with distance ratios (**b**) 0.5, (**c**) 0.4, (**d**) 0.3 and (**e**) 0.2 for an underground dam height equal to 28 m.

At the steady-state condition, the results of the seawater intrusion showed a good comparison between the SEAWAT results and the previously estimated value (Fig. 19.a). The length of the seawater wedge equalled 979.2 m in the steady-state condition, in contrast with 984.6 m in Armanuos et al. ^46^. After reaching the steady-state condition, the built model was utilised to simulate various scenarios, including the embedment of the underground dam with a distance ratio X_d_/L_o_ = 0.2, 0.3, 0.4 and 0.5. For each barrier wall distance, four different underground dam heights were examined: 25, 28, 31 and 34 m, with a total of sixteen configurations. Embedment of the underground dam in the Akrotiri coastal aquifer caused the retreat of the seawater intrusion and a reduction in the seawater intrusion length ratio. For most cases, the underground dam effectively hindered the seawater intrusion and resulted in a full removal of the SI behind it, Figs. 18 and 19.

Figure 19 represents the steady-state SWI distribution for the Akrotiri coastal aquifer, Cyprus, after the embedment of the underground dam with a height equal to 28 m with distance ratios X_d_/L_o_:(a) 0.5, (b) 0.4, (c) 0.3 and (d) 0.2. The underground dam was successful in mitigating seawater intrusion in most cases. For the case with a underground dam distance ratio equal to 0.2, the saltwater removal efficiency reached approximately 60% due to the relatively small distance between the dam and the seawater boundary.

**Fig. 20:**
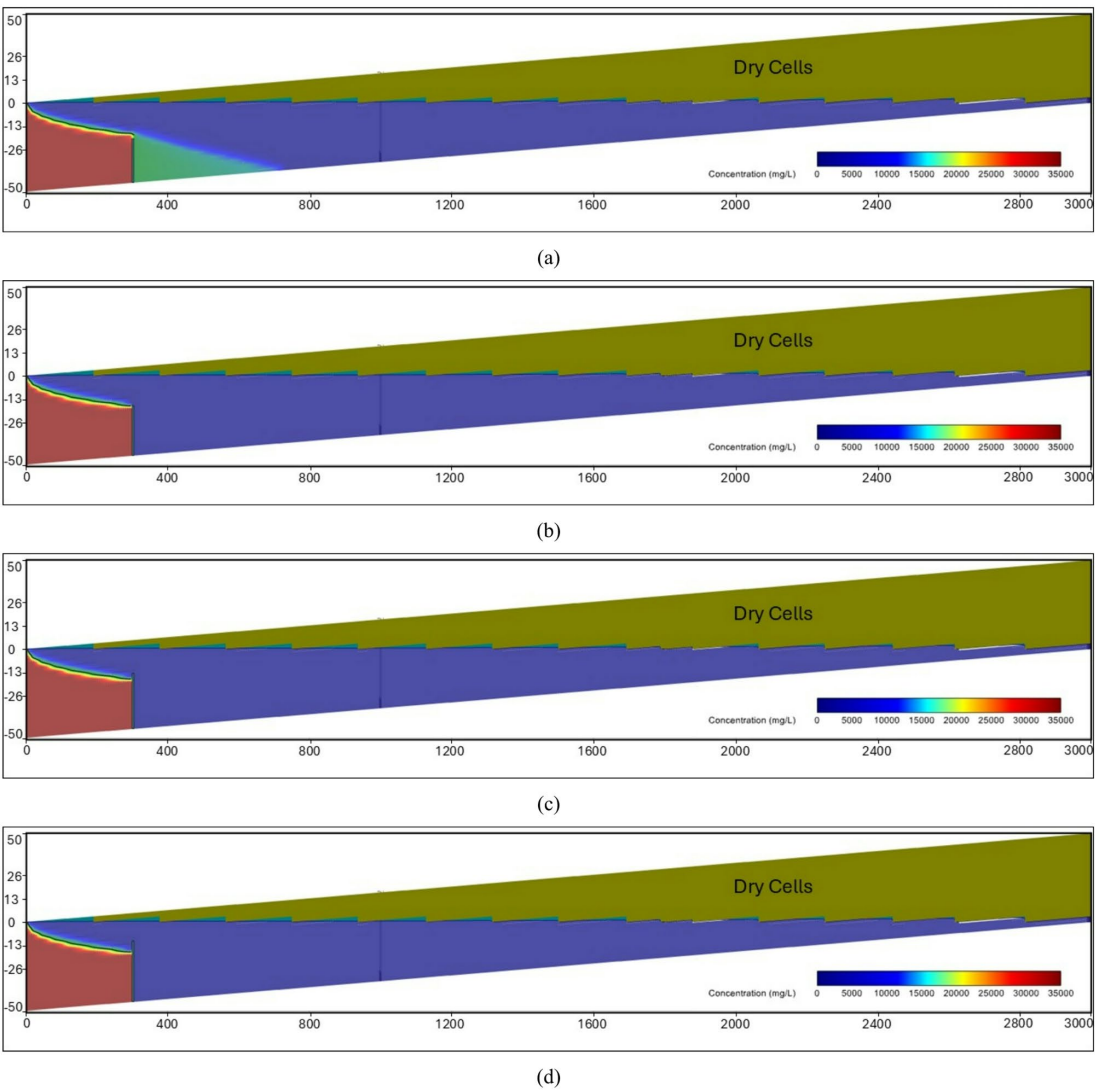
Steady-state seawater intrusion distribution for the Akrotiri coastal aquifer, Cyprus, after the embedment of an underground dam with distance ratios equal to 0.3 for underground dam heights equal to (**a**) 25, (**b**) 28, (**c**) 31 and (**d**) 34 m.

Figure 20 shows the impact of the embedment of underground dams with distance ratios equal to 0.3 for an underground dam height (H_d_/H) equal to (a) 25, (b) 28, (c) 31 and (d) 34 m. For all cases, the underground dam was able to remove the residual seawater intrusion.

**Fig. 21:**
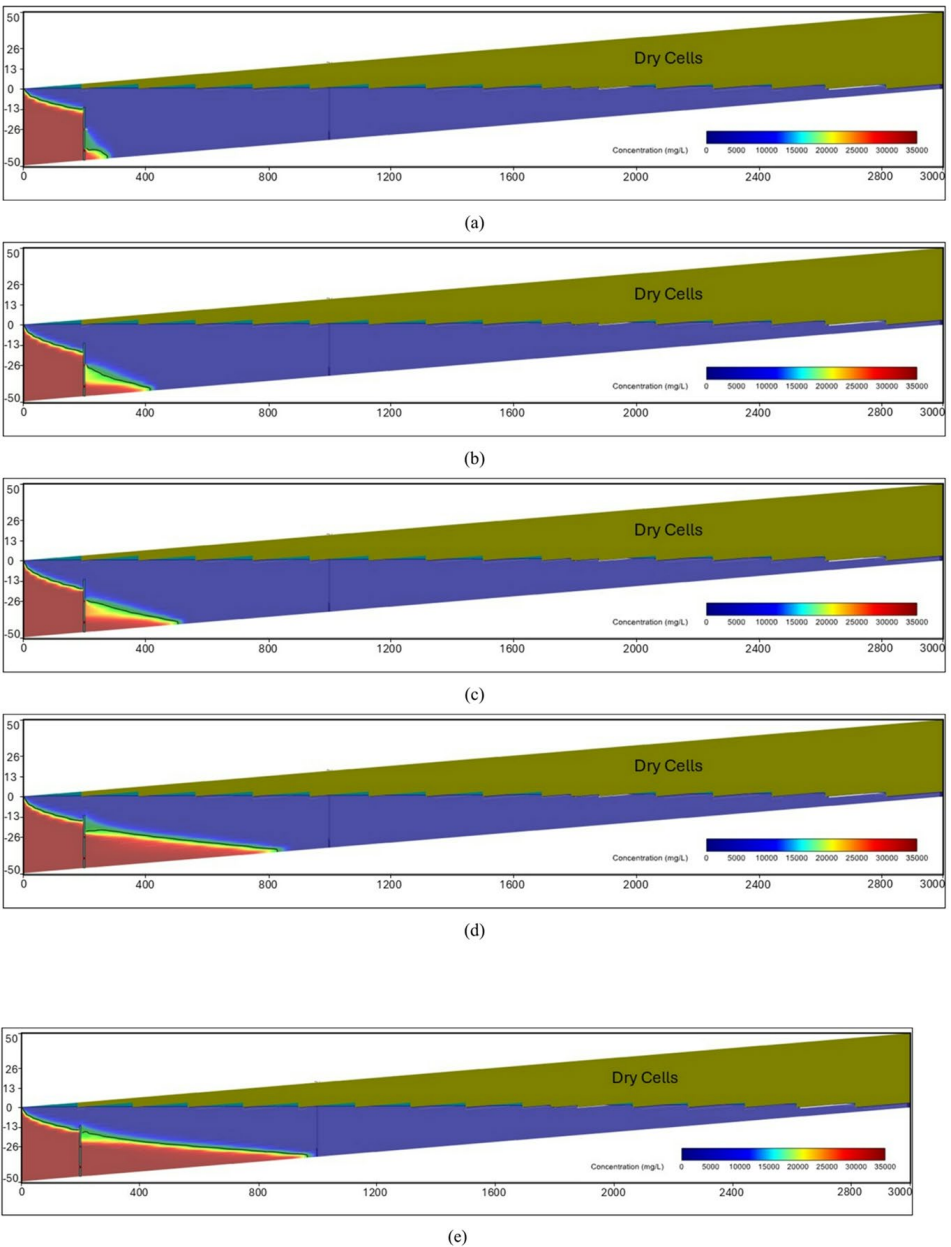
Transient saltwater intrusion distribution for the Akrotiri coastal aquifer, Cyprus, through two fractures in an underground dam with distance ratios equal to 0.2 for underground dam height equal 34 m, for T = 1 year (**a**), T = 5 years (**b**), T = 10 years (**c**), T = 50 years (**d**) and T = 100 years (**e**).

Figure 21 shows the transient saltwater intrusion distribution for the Akrotiri coastal aquifer, Cyprus, through two fractures in a underground dam with distance (X_b_/L_o_) ratios equal to 0.2 for underground dam height equal 34 m, for T = 1 year (a), T = 5 years (b), T = 10 years (c), T = 50 years (d) and T = 100 years (e).

**Fig. 22:**
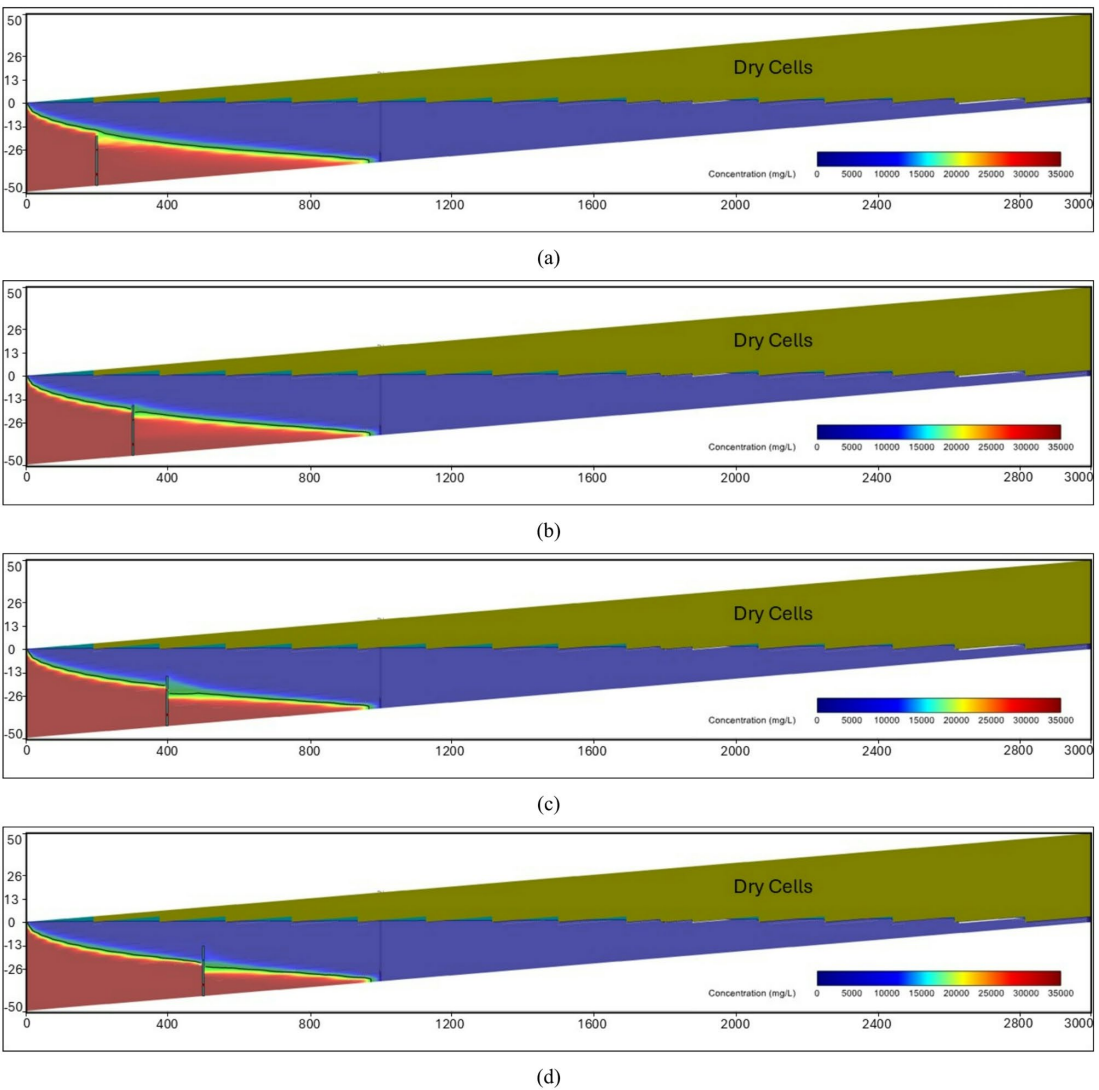
Steady-state saltwater intrusion distribution for the Akrotiri coastal aquifer, Cyprus, for double-fractured underground dams with distance ratios of (**a**) 0.2, (**b**) 0.3, (**c**) 0.4 and (**d**) 0.5 for an underground dam height equal to 28 m.

Figure 22 represents the steady-state saltwater intrusion distribution for the Akrotiri coastal aquifer, Cyprus, for a double-fractured underground dam with a height equal to 28 m with distance ratios of (a) 0.2, (b) 0.3, (c) 0.4 and (d) 0.5. For all cases, the seawater intrusion invaded the aquifer, reaching the original steady state intrusion toe length.

**Fig. 23:**
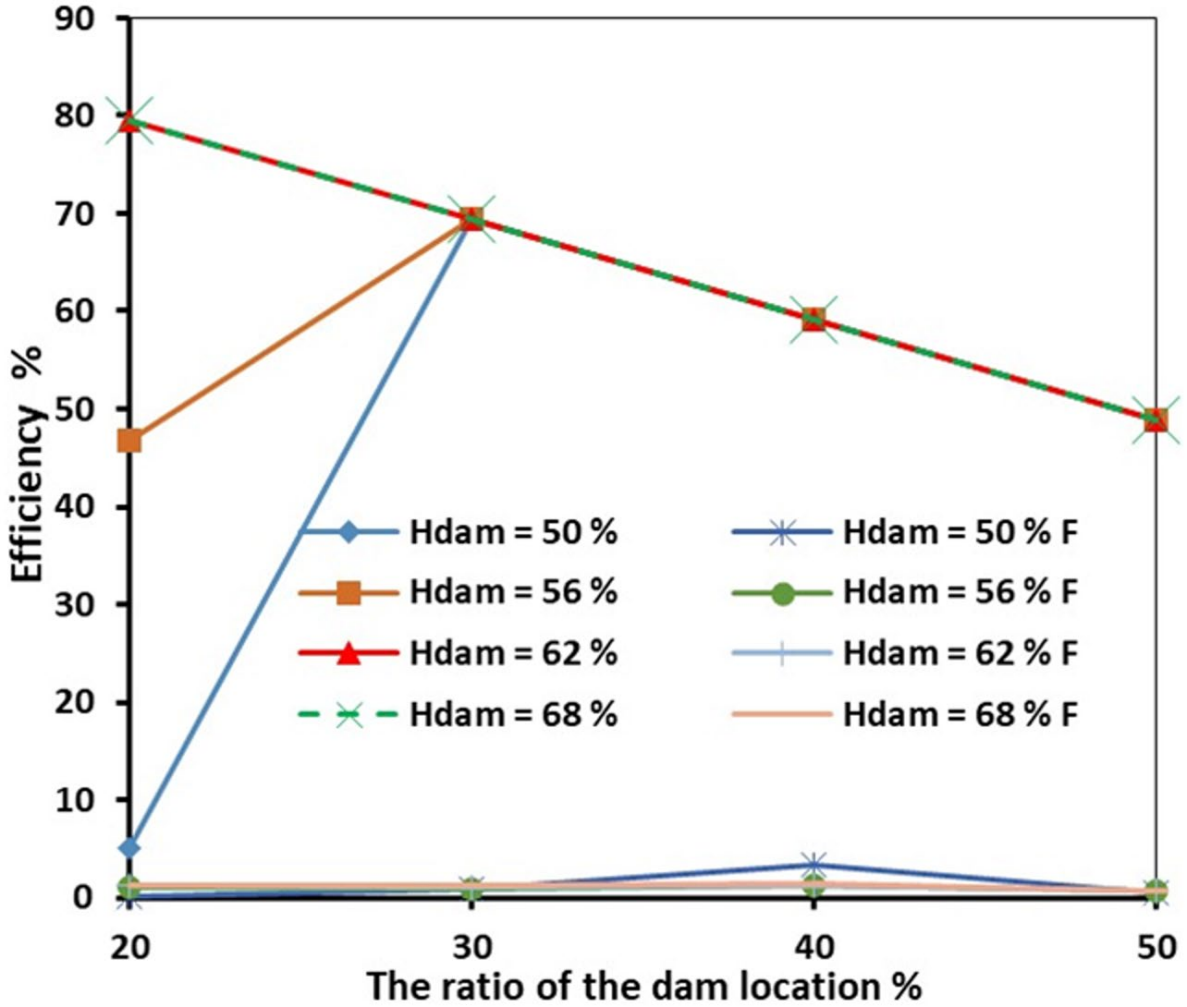
The relation of the underground dam location and the dam height ratio, with the underground dam efficiency in mitigating saltwater intrusion for the Akrotiri coastal aquifer, Cyprus, for both intact and double-fractured underground dams.

The dam’s location was evaluated in four different configurations, and the impact of varying its height was also analysed in four scenarios with a total of 16 configurations. These assessments covered all relevant cases within the Akrotiri coastal aquifer, Cyprus. The findings demonstrated that the dam consistently performed effectively in mitigating saltwater intrusion across most of the tested locations and elevations. Figure 23 illustrates the dam’s efficiency as a function of its position and depth. On average, the dam reduced seawater intrusion by approximately 57%, highlighting its high effectiveness in controlling saltwater encroachment. For the cases with the double-fractured underground dam, the efficiency of the underground dam in mitigating saltwater intrusion plummeted to 1.13%, with an average reduction in efficiency of about 98%.

This study delivers valuable insights into the impact of groundwater abstraction on the efficiency loss of a double-fractured underground dam in saltwater intrusion mitigation; however, limitations should be acknowledged. The simulations were performed using a deterministic numerical model (SEAWAT). Probabilistic modelling and uncertainty analysis were not included, which restricts how broadly the findings may be used. Future work should incorporate stochastic approaches to better describe parameter variations and measure prediction uncertainty. The groundwater aquifer system was characterised using the Henry problem, which assumes that the aquifer is isotropic and homogenous. Real case studies of coastal aquifers are typically heterogeneous and anisotropic, which stratified layers and variable hydraulic properties; thus, incorporating real hydrological settings would improve the applicability of the outcomes. The double fractures in the underground dam were implemented as a simplified horizontal. In real cases, cracks and fractures develops into interconnected networks with complex geometries. The model simulations were performed in two dimensions. Extending the analysis in three dimensional models would produce more reliable predictions. Diffusion and dispersion coefficients were adjusted according to simplified assumptions, which may vary from the real conditions. Validations across laboratory experiments or field case studies are vital to enhance confidence in the model outcomes. Therefore, integrating uncertainty analysis, realistic hydrological heterogeneity, sophisticated future modelling, three-dimensional simulations and experimental validation should be the main goals of future research. Such improvements would support the reliability predictions and enhance the design of more secure underground dam for seawater intrusion control.

## Conclusions

This research presents a comprehensive evaluation of how double-fractured groundwater dams perform against seawater intrusion, with specific emphasis on the impacts of fracture geometry, dam parameters and groundwater extraction. Using SEAWAT code applied to both the Henry problem and the Akrotiri Coastal Aquifer in Cyprus, the research systematically evaluated how variables such as fracture height and aperture, opening, dam location and depth and pumping intensity affect dam effectiveness. The outcomes clearly show that underground dams positioned close to the shoreline are more susceptible to efficiency losses. When the dam location ratio (X_d_/L_o_) was small, the dam suffered significant performance declines, while moving the dam further inland (X_d_/L_o_=0.8) resulted in a significant reduction in efficiency losses. The fracture height also emerged as a critical factor. A fracture close to the dam base delivers preferred pathways for dense saltwater to bypass the dam, expanding the seawater wedge and significantly declining its effectiveness. In contrast, a fracture located at higher height had less pronounced effects. Also, fracture aperture significantly affected saline intrusion: wider fractures allowed greater amounts of seawater to flow upstream, increasing efficiency losses. This suggests that even minor differences in fracture geometry can lead to significant performance differences, underscoring the importance of detailed geological characterisation prior to construction. Groundwater withdrawal exacerbates the problem. As abstraction rates rise, the loss of efficiency increases proportionally, with sharp declines detected in all well height ratios tested scenarios. High pumping essentially draws saltwater more forcefully through the cracks, undermining the dam’s protective role. Therefore, managing the extraction is just as important as designing the dam itself, as sustainable extraction rates must be carefully determined to maintain dam performance.

The research also investigated the role of seawater density. While higher densities lengthened the seawater wedge and increased efficiency loss, for transient simulations, the efficiency loss increased with both seawater density and the ratio X_w_/X_d_. For a density of 1022 kg/m³, the loss rose compared with the base case (a dam without a double fracture) by about 20, 40, 60 and 80% at X_w_/X_d_= 0.8, 1.0, 1.2 and 1.4, respectively.

The current study concluded that the presence of two fractures in an underground dam, in addition to the presence of an abstraction well, reduces the efficiency of the dam in controlling SWI more than the case of the presence of single fracture only and the case of a single fracture with the presence of an abstraction well. The efficiency loss for a double-fractured dam ranged from 27.5% to 140%, and from 14.7% to 63% for a single-fractured dam without an abstraction well, and from 8.75% to 123% for a single-fractured dam with an abstraction well, under the same conditions. For all cases, at the Akrotiri coastal aquifer, Cyprus, the seawater intrusion invaded the aquifer, reaching the original steady-state intrusion toe length. This research recognises key parameters affecting the underground dam efficiency and introduces practical recommendations. To sustain long-term efficiency, an underground dam should be positioned at a safe distance from shorelines, as proximity accelerates performance drop. Equally important is evaluating fractures, especially those close to the dam bottom, as they can significantly impair dam performance to mitigate SWI. Abstraction rates must also be wisely managed, particularly in fractured systems, whereas fracture aperture size needs attention, as greater apertures accelerate efficiency losses. While the study sheds light on the efficiency of underground dams in mitigating saltwater intrusion, it also notes several limitations. The fracture models used were based on specific sizes and arrangements, which may not reflect the full diversity of real-world fracture patterns. In practice, fractures can differ greatly in their dimensions, shapes and angles – all factors that influence how well a dam performs. To strengthen future work, researchers should include thorough economic evaluations, such as cost-benefit analyses and assessments of construction and maintenance challenges. A more comprehensive approach will support better decisions about the feasibility and long-term sustainability of underground dam projects.

## Data Availability

Data are available on the request to the corresponding authors.
